# Drug-based therapeutic strategies for COVID-19-infected patients and their challenges

**DOI:** 10.2217/fmb-2021-0116

**Published:** 2021-11-23

**Authors:** Khatereh Zarkesh, Elaheh Entezar-Almahdi, Parisa Ghasemiyeh, Mohsen Akbarian, Marzieh Bahmani, Shahrzad Roudaki, Rahil Fazlinejad, Soliman Mohammadi-Samani, Negar Firouzabadi, Majid Hosseini, Fatemeh Farjadian

**Affiliations:** ^1^Pharmaceutical Sciences Research Center, Shiraz University of Medical Sciences, Shiraz, Iran; ^2^Department of Pharmaceutics, School of Pharmacy, Shiraz University of Medical Sciences, Shiraz, Iran; ^3^Department of Clinical Pharmacy, School of Pharmacy, Shiraz University of Medical Sciences, Shiraz, Iran; ^4^Department of Pharmacology & Toxicology, School of Pharmacy, Shiraz University of Medical Sciences, Shiraz, Iran; ^5^Department of Manufacturing & Industrial Engineering, The University of Texas Rio Grande Valley, Edinburg, TX 78539, USA

**Keywords:** antibiotics, antimalarial, antiparasitic, corticosteroids, COVID-19, immunomodulatory drugs, immunosuppressants, therapeutic drugs

## Abstract

Emerging epidemic-prone diseases have introduced numerous health and economic challenges in recent years. Given current knowledge of COVID-19, herd immunity through vaccines alone is unlikely. In addition, vaccination of the global population is an ongoing challenge. Besides, the questions regarding the prevalence and the timing of immunization are still under investigation. Therefore, medical treatment remains essential in the management of COVID-19. Herein, recent advances from beginning observations of COVID-19 outbreak to an understanding of the essential factors contributing to the spread and transmission of COVID-19 and its treatment are reviewed. Furthermore, an in-depth discussion on the epidemiological aspects, clinical symptoms and most efficient medical treatment strategies to mitigate the mortality and spread rates of COVID-19 is presented.

The current epidemic of SARS-CoV-2, the virus that induces illness known as COVID-19, is an important wake-up call for all countries around the world. Given the dire global consequence that outbreaks of emerging diseases like COVID-19 can trigger, there is an urgent need to improve preparedness for such epidemic-prone diseases. COVID-19 is not the first global pandemic, and emergence of new epidemic-prone diseases is inevitable due to poor humanitarian habits.

Since the start of this century, large outbreaks such as COVID-19, Ebola virus, MERS-CoV, SARS-CoV-2 and influenza A virus subtype H1N1 (A/H1N1) have shattered several myths about the global vulnerability to threats arising from such epidemic-prone diseases [1,2]. For instance, COVID-19 proved how quickly a new virus could spread around the world. Several broad-scale factors contributed to the quick dissemination of COVID-19 in its early phases, including delays and shortage of vaccines, lack of following quarantine procedures, viral viability at different climates, viral transmission through domestic and international travel, changing diets and human population growth. These factors have amplified human exposure rates to COVID-19. Although growing vaccination rates, lockdowns and social distancing have assisted in reducing the spread of COVID-19 in many countries, lockdown has also negatively impacted the global economy. Although the disease outlook for COVID-19 is more favorable than SARS or H1N1, the number of deaths and confirmed cases of COVID-19 exceeds that of SARS or H1N1, which makes COVID-19 harder to manage in comparison with other viruses.

SARS-CoV-2 (order Nidovirales, family Coronaviridae, subfamily Coronavirinae) is a virus that contains a single-stranded ribonucleic acid genome and a nucleocapsid phosphoprotein (Figure 1). Binding of SARS-CoV-2 to angiotensin-converting enzyme 2 (ACE2) enables viral entry to the cell and causes many respiratory diseases in human beings and various ranges of diseases in animals [3].

**Figure 1.**
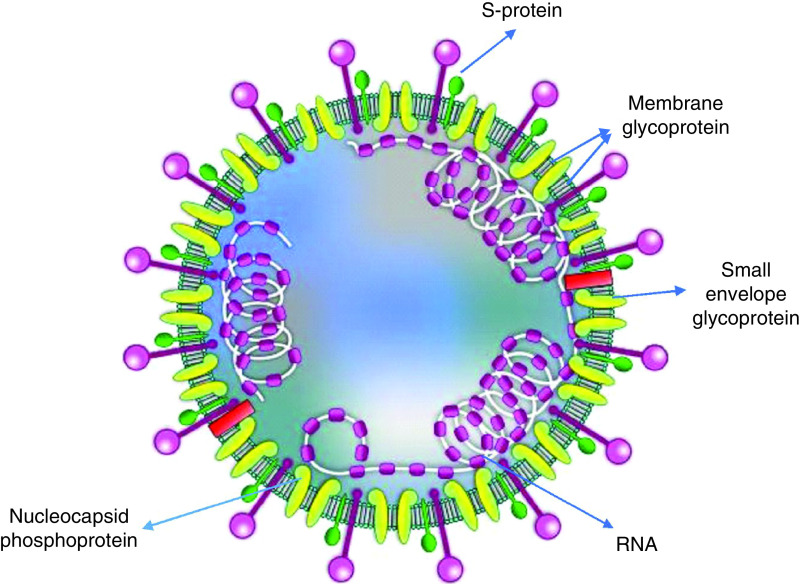
Structure of coronavirus and its components. The coronavirus is a virus that contains ribonucleic acid genome and nucleocapsid phosphoprotein, which causes many respiratory diseases in human beings and various ranges of diseases in animals. Spike glycoprotein (S-protein) of the coronavirus represents the primary pattern in entering the host cells.

SARS-CoV-2 can target human cells by the binding of spike glycoprotein (S-protein) to ACE2 receptor, a membrane-bound carboxypeptidase (Figure 2). ACE2 has a C-terminal collectrin-like domain and an N-terminal peptidase domain. ACE2 receptors are predominantly located in the apical surface of the lung airway epithelium and enterocytes of the small intestine [4].

**Figure 2.**
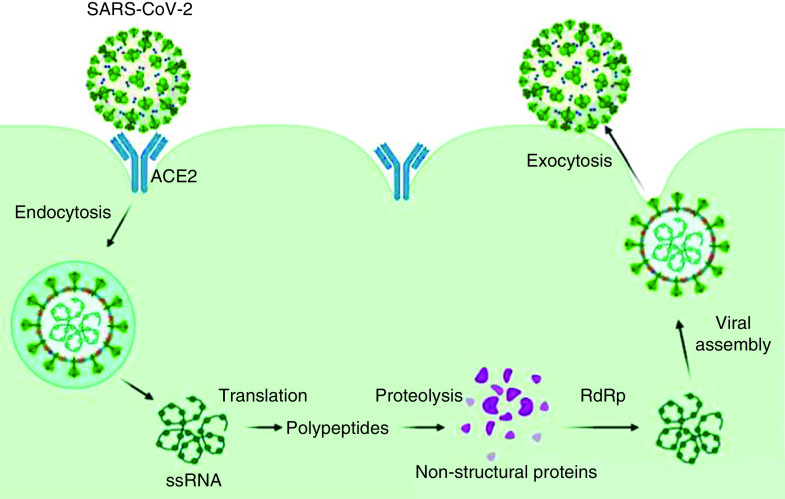
Schematic view of SARS-CoV-2 lifecycle stages and mechanism of action. SARS-CoV-2 can target human cells via the binding of spike glycoprotein to ACE2 receptor, which is a membrane-bound carboxypeptidase. After the binding of SARS-CoV-2 to ACE2 receptors, endocytosis takes place through cell receptors and forms endosomes. Then, the viral uncoating process results in RNA exit from the viral structure. Translation of ssRNA results in the synthesis of viral polypeptides that undergo the proteolysis process and form non-structured proteins. These polyproteins can encode the RTC. After that, SARS-CoV-2 synthesizes the ssRNA via the RdRp enzyme. Then, translation results in the fabrication of structural proteins. Finally, viral assembly and viral release through exocytosis take place. ACE2: Angiotensin-converting enzyme 2; RdRp: RNA dependent RNA polymerase; RTC: Replicase-transcriptase complex.

Since ACE2 acts as an entry pathway for viral infection, blocking this receptor through ACE inhibitors and receptor blockers is promising to prevent viral infection into the lungs and whole body. Also, it has been suggested that the implementation of human recombinant ACE2 (hr-ACE2) protein would be helpful to saturate viral S- proteins and prevent SARS-CoV-2 cell entrance [4].

According to Figure 2, the binding of SARS-CoV-2 to ACE2 receptors stimulates endocytosis through cell receptors and subsequently forms endosomes. The viral uncoating process via shedding of the endosome results in RNA exit from the viral structure. Translation of ssRNA results in the synthesis of viral polypeptides that undergo the proteolysis process and form non-structured proteins. Recent studies have reported that lopinavir and darunavir are able to interfere with virility at this stage. These polyproteins encode the replicase-transcriptase complex (RTC). Subsequently, SARS-CoV-2 synthesizes the ssRNA via the RNA dependent RNA polymerase (RdRp) enzyme. Then translation results in the fabrication of structural proteins. Finally, viral assembly and release through exocytosis occur [5]. The schematic view of the SARS-CoV-2 lifecycle stages and mechanism of action is depicted in Figure 2.

Padhi and Tripathi have explored putative drug binding sites in the main protease of SARS-CoV-2. They report mutation hot spots that may explain probable drug resistance [6]. This team also reported mutations in the S-protein domain that could reduce the effectiveness of therapeutic agents and increase the infectivity and pathogenicity of the virus [7].

Coronaviruses are categorized into four subgroups: alpha, beta, delta and gamma. Only alpha and beta may be transmitted to humans and therefore are characterized as human coronaviruses (HCoVs) [8].

In late 2019, new cases of beta coronavirus infections were identified in Wuhan, China. Due to the acute respiratory symptoms of this recently recognized coronavirus, it was subsequently named SARS-CoV-2, and the WHO named this new coronavirus disease COVID-19.

Patients infected with this virus show different signs and symptoms in the following organ systems: respiratory, neurological, gastrointestinal and cardiovascular. The more prevalent symptoms include cough, sore throat, fever, fatigue, headache, arthralgia, anosmia and ageusia [9].

In less than 14 months from the first reported cases, the virus spread globally. The pandemic became one of the world's leading challenges in 2020, with a major impact on the economy and more than 5 million mortalities worldwide.

Many efforts have been made to develop an efficient vaccine for this viral infection. The main types of COVID-19 vaccines could be categorized as mRNA [10,11], viral vectors [12] and protein subunits [13]. To date, at least three different COVID-19 vaccines have been authorized in the USA, including Moderna COVID-19 vaccine (mRNA-1273), Pfizer-BioNTech COVID-19 vaccine (BNT162b2) and Johnson & Johnson COVID-19 vaccine (JNJ-78436735, formerly Ad26.COV2. S). Other COVID-19 vaccines have also been developed and authorized in other countries, including AstraZeneca COVID-19 in the UK (AZD1222, also known as Covishield); CoronaVac, BBIBP-CorV and Convidicea (Ad5-nCoV) in China; Sputnik V, EpiVacCorona and CoviVac in Russia; Covaxin in India; and COVIran Barakat in Iran.

In addition to the vaccines mentioned above, more than 200 additional vaccine candidates are currently in the development phase, of which more than 60 are under clinical investigation [14]. However, various levels of efficacy have been reported (i.e., from 50% to 95%). Different approaches to vaccine design and technologies are likely the culprit. Vaccine distribution started in December 2020. Manufacturing and supplying sufficient doses of vaccines for 7.8 billion people worldwide, the duration of vaccine protection and vaccine efficiencies are some of the challenges for healthcare providers worldwide. Additionally, new mutants of COVID-19 with enhanced virility have been observed, especially in South Africa, India, Brazil and the UK. These new mutants may allude to the immunity encouraged by the vaccines developed to target initial strains of the SARS-CoV-2 virus.

As previously mentioned, emerging epidemic-prone diseases in the 21st century have introduced numerous health and economic challenges, resulting in considerable mortality of the human population. To control and mitigate COVID-19 and address the immediate and long-term health outcomes, it is imperative to have comprehensive understanding of the nature of COVID-19 evolution, its mode of action and transmission, the potential ecological factors influencing its emergence (i.e., whether it represents the re-appearance of a familiar and previously studied pathogen) and its potential connection to previous events.

As the delta variant of the coronavirus spreads throughout the world, it is essential to develop efficient therapeutic strategies to protect infected patients against severe illness, hospitalization and death. A guide to understanding the limiting factors for large-scale trials of therapeutic strategies for COVID-19 treatment albeit the spread of the delta variant and vaccine hesitancy and refusal is highly encouraged. Given the rapid spread of the delta variant, and the worldwide economic loss, it is imperative to develop effective therapeutic drugs to treat COVID-19 patients. Significant research efforts have been invested in the development of therapeutic drugs to treat COVID-19 patients. It has been a challenging process communicating pandemic-related information, especially amid misinformation, disinformation, conspiracy theories and politicization. As a result, health communicators are facing difficulties in coming across as credible, transparent and trustworthy. The delta variant has also dramatically upended recent progress against the pandemic and is forcing a reset everywhere. In this review article, the authors summarize the current understanding of COVID-19, such as its epidemiological aspects, clinical symptoms, pathophysiology and pharmacologic categories, as well as the most efficient medical treatment strategies supported by the NIH and WHO to help reduce severity and mortality rates. The potential mechanisms of action, dosing, adverse drug reactions and contraindications are also discussed in detail. Further, the most recent information about COVID-19 transmission and prevention is presented. Finally, research insights, existing knowledge gaps, challenges and future research direction are described, and a state-of-the-art snapshot of drug-based therapeutic strategies for COVID-19 and the attempts to repurpose various classes of drugs is presented.

## Epidemiology

Compared with SARS-CoV, COVID-19 has a robust transmission capacity [15]. Patients with severe symptoms correspond to enhanced viral spread compared with patients with milder symptoms. Asymptomatically infected individuals and patients who display no signs of respiratory complications are other potential sources of infection. COVID-19 is a self-limiting illness, and the recovery period in most patients with mild symptoms lasts 1–2 weeks [16]. Epidemiological evidence indicates this disease transmits through humans by the basic reproductive number of 2.2 [17]. It seems that the mortality rate is higher in men than women (4.7% vs 2.8%), and the fatality rate worsens in patients older than 60 years (3.6%) compared with individuals younger than 40 years (0.2%). Furthermore, patients with pre-existing comorbidities such as diabetes, hypertension and cardiovascular diseases show higher fatality rates [18,19].

It is noteworthy that epidemiological trends in the outbreak and fatality rate of COVID-19 vary on a daily basis and new mutants of the virus were reported in Africa, Brazil and the UK. At the beginning of the pandemic, China was leading in several diagnosed cases. Still, the incidence progressively increased in other countries, mainly Europe, the USA and the Middle East [20,21].

Among the diseases caused by coronaviruses, SARS, MERS and COVID-19 are most prevalent, resulting in severe outcomes in humans. Herein, the root of such diseases and their symptoms are discussed.

### SARS

In 2003, SARS was transmitted to humans and other animals (civet cats) by bats. The first infected human was diagnosed in Guangdong Province, China. Since then, the virus has spread to 26 countries and resulted in more than 8098 cases, with a fatality rate of 9.6% [22].

Like those of influenza, the symptoms included fever, malaise, myalgia, headache, diarrhea and chills (rigors). In the first and second weeks of illness, patients experienced dry cough, shortness of breath, gasp and diarrhea. In severe cases, the disease often progressed rapidly, leading to respiratory failure and requiring intensive care, such as supplementary oxygen [23].

### MERS

MERS was first diagnosed in Saudi Arabia in September 2012. It infected 124 people and caused 52 deaths. The clinical manifestations of this disease were respiratory problems such as sore throat, dry cough and dyspnea. The mortality rate was approximately 60% [24]. In 2018, MERS was transmitted to the Republic of Korea by a passenger traveling to Kuwait and caused a MERS-CoV outbreak in South Korea [25].

### Clinical Symptoms of SARS-CoV-2

Although clinical symptoms are important in diagnosis, no individual symptom has been found to diagnose SARS-CoV-2. In less than a week, the symptoms include fever, nasal congestion, cough and fatigue, and other signs related to upper respiratory infections appear [26]. Some references describe asymptomatic infections and gastrointestinal (GI) symptoms, especially in young children [27].

In addition to the respiratory manifestations of COVID-19, other complications such as neurologic [4], psychiatric, ophthalmologic, hepatobiliary and cutaneous presentations have been observed [28]. According to different studies on COVID-19 patients, cardiovascular manifestations, including arrhythmia, myocarditis, myocardial ischemia and myocardial infarction (MI; type 1 and 2), cardiomyopathy and cardiogenic shock, have also been observed [18,29]. Among the neurologic and ophthalmologic symptoms, anosmia, ageusia, anorexia, fatigue, headache, dizziness, stroke, myalgias, conjunctivitis and encephalopathy have been reported [9,30–32].

The most common dermatological manifestations presented in COVID-19 patients are urticaria, erythematous rash, livedo reticularis and petechiae [33,34]. Severe cases progress quickly with respiratory failure due to serious alveolar damage. Moreover, organ dysfunction can occur, such as acute respiratory distress syndrome (ARDS), acute kidney injury, acute cardiac injury, shock and death [35].

In a study on patients with mild infection, abnormal laboratory results were obtained, such as increased levels of the erythrocyte sedimentation rate (ESR), C-reactive protein (CRP), D-dimer and lactate dehydrogenase (LDH) [34,36]. Studies on patients with severe infections have revealed similar results, including increased values of white blood cells (WBCs), aspartate aminotransferase (AST), LDH, creatinine, cardiac troponin, D-dimer and prolactin [37]. Increases in the levels of leukocytes, neutrophils, prothrombin (PT) and alanine aminotransferase (ALT) have also been reported. However, the values of lymphocytes and albumin decreased to some extent [38,39]. Standard laboratory tests for COVID-19 are summarized in Table 1.

**Table 1.** Common laboratory test results of complete blood counts and blood chemistry results for COVID-19 patients, categorized according to increased and decreased levels.

Increased	Decreased
WBC (1.5-×)	Lymphocyte count (0.4×)
Neutrophil count (4.4×)	Albumin (0.8×)
LDH (2.1×)	
ALT (1.8×)	
Total bilirubin	
AST (1.5×)	
CRP	
Procalcitonin (1.2×)	
PT (1.4×)	
Creatinine (1.1×)	
D-dimer (2.5×)	
Cardiac troponin (2.2×)	

ALT: Alanine transaminase; AST: Aspartate transaminase; CRP: C-reactive protein; LDH: Lactate dehydrogenase; PT: Prothrombin; WBC: White blood cells.

### Breakthrough COVID-19 cases

A concerning point in this illness is the possibility of reinfection. Vaccines can stop most people, but not everyone, from getting sick with COVID-19. Despite having been fully vaccinated, there is still a chance that people can get infected.

Tillett *et al.* [40] reported the first COVID-19 reinfection case of a 25-year-old male who had recovered from COVID-19 and had two consecutive reverse transcription polymerase chain reaction (RT-PCR) tests after the recovery period. After 48 days from his first positive RT-PCR diagnostic test, he had another positive RT-PCR test. A SARS-CoV-2 genome sequencing study revealed that both specimens were genetically the same in terms of their clade (clade 20C) but significantly different in genome sequences. It was reported that the SARS-CoV-2 reinfection was worse than the first infection and resulted in oxygen requirement and hospitalization [40]. However, it should be noted that for the majority of breakthrough cases, patients have experienced mild, “cold-like” symptoms.

To date, the association between immunodeficiency and SARS-CoV-2 reinfections has not been confirmed. The exact incidence of SARS-CoV-2 reinfection is not clear and, given the vast number of asymptomatic cases, this rate is likely underreported [41]. The most important point in the differential diagnosis of SARS-CoV-2 reinfection versus SARS-CoV-2 reactivation would be viral genome sequencing. The former can result in different viral genome sequences, and the latter had the same genome sequences [41]. In order to assay the association between SARS-CoV-2 reinfection and immune response after the first COVID-19 infection, the level and specificity of anti-S protein antibody at the time of reinfection should be evaluated [41].

## Overview of proposed COVID-19 therapeutic drugs

Despite all efforts, the mortality rate related to COVID-19 is still high. Researchers worldwide are investigating alternative approaches to fighting the COVID-19 global pandemic. Herein, putative therapeutic agents under investigation for the management of COVID-19 are discussed. Therapeutic agents of various categories—including antiviral, antimalarial, antiparasitic, anti-inflammatory, mucolytic, immunomodulator, corticosteroid, immunosuppressant, anticoagulant and cardioprotective drugs—have been prescribed to treat COVID-19 patients and were shown to be efficacious in their recovery. This review will be a new collection in COVID-19 treatment in continuation of the previous efforts of Farjadian *et al.* in writing review articles on various pharmaceutical topics [42–48].

### Antiviral agents

#### Favipiravir

Favipiravir is a purine nucleic acid analog [49] that is converted to the acting form of favipiravir ribofuranosyl-5′-triphosphate (T-705-RTP) *in vivo* [50]. It is widely used to treat influenza A and B, Ebola virus, arenavirus, bunyavirus, flavivirus and filoviruses [51]. It is an inhibitor of viral RdRp and widely administered in the treatment of COVID-19 [52]. A recent *in vitro* study reported that favipiravir demonstrated an inhibitory effect on COVID-19 [18].

A clinical trial on 35 patients receiving favipiravir compared with LPV/ritonavir was performed on COVID-19 patients. The first group demonstrated a faster recovery period and shorter clearance time of the virus than the control group [53]. However, another randomized clinical trial showed that patients who received a 7-day course of favipiravir had no beneficial clinical symptoms compared with patients of umifenovir but those did improve clinical symptoms such as cough [54].

#### Lopinavir/ritonavir

These are protease inhibitors that were first approved in 2000 to prevent HIV infection. Ritonavir also raises the plasma levels of lopinavir (LPV) by inhibiting cytochrome p 450 (CYP450) [55,56]. With the outbreak of the SARS virus in 2003, this combined medicine under the brand name of Kaletra^®^ showed a viral inhibitory effect in *in vitro* studies. The outcomes of LPV-ritonavir treatment for COVID-19 patients were investigated [57]. The administration of this drug did not display a significant clinical improvement in comparison with that of the standard group [57].

#### Remdesivir

Remdesivir was first discovered by Gilead Sciences Company [58]. Also, remdesivir demonstrated a prophylactic effect on MERS-CoV infection in rhesus monkeys [59]. Remdesivir is an analog of a nucleotide monophosphate prodrug that is converted to the active form of remdesivir C-adenosine nucleoside triphosphate analog [60]. It attaches to the RNA polymerase of the virus, interrupting transcription of the virus and inhibiting RdRp enzyme activity [61,62]. An *in vitro* study in Vero E6 cells demonstrated the antiviral effects of remdesivir (GS-5734) against COVID-19. Remdesivir was shown to be capable of inhibiting the virus with higher sensitivity and at a lower concentration [18].

In a study conducted on patients from the US, Europe, Canada and Japan, remdesivir improved clinical manifestations [63]. A recent case study of patients showed that intravenous injection of remdesivir improved patients' recovery time and mortality compared with that of placebo [64]. In another study, the duration of intravenous remdesivir administration for 5 and 10 days was investigated [65]. No significant difference in the improvement of clinical symptoms for 5- or 10-day administration was reported [65].

#### Ribavirin

Ribavirin is another RNA antiviral drug. It received US FDA approval in 1998 to treat chronic hepatitis C (CHC). According to the primary case study in the USA, the flagship study on ribavirin did not show more benefits in treating patients than LPV/ritonavir. However, new clinical studies have shown therapeutic effects on patients [66,67]. In general, this drug may be considered a possible treatment for COVID-19, based on its performance in prior studies on similar diseases [68].

#### Sofosbuvir

Sofosbuvir is an antiviral that directly targets the RNA of the virus. It is used as a combinatorial therapy with ribavirin and interferon alpha (IFN-α) [69] to treat hepatitis C virus (HCV). Prior studies have suggested the curative effect of sofosbuvir in COVID-19 infections. Molecular modeling and an *in vitro* study have also confirmed its role in COVID-19 [70,71].

#### Umifenovir

Umifenovir is an antiviral drug utilized as a prophylaxis for influenza A and B viruses in Russia and China for several decades [72]. Umifenovir acts by interfering with the virus's interaction with host cells, likely by binding to ACE2 receptors [73].

A clinical study reported that the administration of umifenovir (Arbidol^®^) in comparison with LPV/ritonavir could be effective, as seen by faster viral clearance compared with patients who received LPV/ritonavir. Based on the reports in a 14-day period, all patients receiving Arbidol^®^ had a negative viral load, whereas the patients in the LPV group had a positive viral load [69].

### Antimalarial agents

#### Hydroxychloroquine

Chloroquine (CQ) is the standard of care to prevent malaria (*Plasmodium falciparum*) infection. However, CQ is limited by an increased risk of high-grade side effects such as cardiac arrhythmias (i.e., QT prolongation). Hydroxychloroquine (HCQ) has been formulated and found to be less toxic (∼40%). HCQ is now prescribed as an immune modulator for autoimmune diseases such as systemic lupus erythematosus (SLE) and rheumatoid arthritis (RA) [74]. An *in vitro* (Vero E6 cells) study revealed that CQ with half-maximum effective concentration (EC50) of 1.13 μm, half cytotoxic concentration (CC50) >100 μm was required to reduce the viral load of COVID-19 [18]. Moreover, another *in vitro* study reported that HCQ had more potent inhibitory effects compared with CQ in SARS-CoV-2. Both CQ and HCQ prevented the entrance of the virus into the cell through an increase in the pH value of the endosome and alteration of the glycoside transferase of the SARS ACE2 receptor [75]. Importantly, an observational study revealed that the combination of HCQ and azithromycin (AZN) enhanced clearance and improved symptoms in 100% of patients [76].

It was reported that patients receiving high doses of CQ diphosphate lowered mortality rate compared with patients who received low doses of CQ diphosphate. However, the high-dosage group demonstrated more toxic effects and lethality compared with the low-dosage group 2M3 [71,77]. In contrast with the result discussed above, QT elongation was not observed. Significant improvements in symptoms and mortality rate were found in those patients who received HCQ alone or the combination of AZN and HCQ [78]. However, another study on hospitalized COVID-19 patients reported that concomitant use of AZN and HCQ led to QT-interval prolongation and increased risk of mortality [79,80].

### Antibiotics

#### Azithromycin

AZN is a macrolide that is efficacious against Gram-negative and Gram-positive bacteria. It has been used to treat respiratory (bronchitis and pneumonia), enteric and genitourinary tract infections [81]. Several *in vitro* studies have recognized that AZN is effective against Ebola and SARS-Cov-2 with EC_50_ values of 2.792 and 2.12 μm, respectively [82]. Furthermore, in an *in vitro* study, concomitant administration of AZN and HCQ resulted in a synergistic effect in COVID-19 treatment [76]. However, to date, clinical proof for the success of AZN in COVID-19 treatment is lacking. AZN exerts its effect through hindering protein synthesis by binding to 50S ribosomal subunit bacteria [77]. However, its antiviral mechanism is not yet known. AZN, like HCQ, increases the pH of the endosome. Macrolides such as AZN, clarithromycin, erythromycin and fidaxomicin also pose anti-inflammatory and immunomodulatory effects by affecting the level of interleukin (IL) (e.g. IL-6, IL-8), tumor necrosis factor, alpha (TNF-α), inhibiting T-helper functions, and reduce the oxidative stress [78]. Cluster of differentiation 147 (CD147) is a transmembrane glycol-protein of the immunoglobulin superfamily that acts as a receptor for *Plasmodium falciparum* and SARS-CoV-2 invasion [83]. AZN could prevent *Plasmodium falciparum* invasion and has shown efficacy in SARS-CoV-2 infection treatment, likely through ligand/receptor interactions [83]. A possible mechanism of AZN action to treat COVID-19 in red blood cells has been reported [83]. By decreasing viral replication and virus release,and increasing levels of IFNs and IFN-stimulated proteins, AZN induces anti-viral responses in epithelial cells [83]. Influenza A and high glucose levels are prompting CD147 expression, which suggests a possible relevance for chronic inflammatory syndromes like diabetes, asthma and the upper level of CD147 in involved patients [83].

In Table 2, the antiviral and antimalarial drugs used in COVID-19 treatment, with descriptions of administration, mechanisms of action, contradictions and major side effects, are briefly discussed.

**Table 2.** Classification of possible COVID-19 treatments in antiviral and antimalarial categories.

Drug	Administration	Mechanism of action	Contraindication	Major side effects	Ref.
Favipiravir	3200 mg-loading doses for 1 day, continued by 1200 mg for 2 to14 days	RNA polymerase inhibitor	Pregnancy and hypersensitivity	Diarrhea, nausea, increased serum transaminases and uric acid decreased in the neutrophil counts	[84]
LPV /ritonavir	LPV/ritonavir 400 /100 mg twice daily for 10 to 14 days	Bind SARS-CoV-2 3C-like proteinase	Hypersensitivity (e.g., Stevens-Johnson syndrome, toxic epidermal necrolysis, urticaria, erythema multiform and angioedema) to any of its ingredients	GI disturbance, headache, liver dysfunction, glycemia and lipid perturbances and renal lithiasis	[79]
Remdesivir	200 mg iv. as loading dose continuous 100 mg for 5 to 10 days	RdRp inhibitors	Hypersensitivity to any ingredient	Diarrhea, vomiting, nausea, rash and liver and kidney injury	[80]
Sofosbuvir	400 mg tablet taken once a day	Nucleotide analog nonstructural protein 5B (NS5B) polymerase inhibitor	Combination therapy for hepatitis C	Fatigue, nausea, headache and insomnia	[85]
Ribavirin	400 mg tablet taken every 12 h	Nucleotide analog NS5B polymerase inhibitor	Combination therapy for chronic hepatitis C	Fatigue, asthenia, headache, rigors, fevers, nausea and myalgia	[57]
Umifenovir (Arbidol^®^)	200 mg/day for 5 to10 days	Inhibit entry into the cell through binding SARS-CoV-2 S-protein	Hypersensitivity to the drug or any ingredient	Elevation of ALT and AST leucopenia	[86]
CQ	300 mg every12 h on day 1, followed by 300 mg × 2 daily for 2–5 days (600–1200 mg for 2–5 days)	Increasing the endosome pH and altering the glycoside transferase	Hypersensitivity to 4-aminoquinoline, psoriasis porphyria retinal or visual field change	Nausea and vomiting, diarrhea, blurring of vision, cardiac toxicity, QT prolongation	[87]
AZN	500 mg orally for 1 day, continued by 250 mg p.o., on days 2–5	Inhibition of protein synthesis, immunomodulatory and anti-inflammatory effects	Hypersensitivity to any macrolides or ketolides, history of cholestatic jaundice and hepatic dysfunction	GI symptom (diarrhea, nausea, vomiting) elevated ALT, AST, creatinine	[88]

ALT: Alanine aminotransferase; AST: Aspartate aminotransferase; AZN: Azithromycine; CQ: Chloroquine; GI: Gastrointestinal; LPV: Lopinavir; QT: prolongation; a measure of delayed ventricular repolarization, RdRp: RNA dependent RNA polymerase.

###  Antiparasitics:

#### Nitazoxanide

Nitazoxanide (NTZ), an antiprotozoal, is the active and oral form of nitrothiazolysalicylamide [89]. It can increase the intracellular production of alpha and beta IFNs. Previous studies have highlighted the antiviral properties of this drug against MERS-CoV [90]. Although the mechanism of action of NTZ is still under investigation, new studies believe that this drug can interfere with viral entry and intracellular proliferation of the Sars-CoV-2 virus. Today, some researchers point to the combination of NTZ/AZN as a more effective treatment for viral infections [91,92]. Some other researchers believe that combining NTZ with HCQ could effectively combat the SARS-CoV-2 virus [93]. It is also suggested that this drug be used in combination with AZN [91]. However, prolonged drug exposure and the toxicity of drug combinations are still not well understood. Nevertheless, the antiviral effect of NTZ against viral infections of HCV, hepatitis B virus (HBV), influenza and coronavirus has been well established. Additionally, there are implications of using NTZ for asthma and therefore potential benefits in treating COVID-19 symptoms [8].

#### Ivermectin

Ivermectin is FDA approved for parasitic infections and tropical diseases. It has been shown that this drug can inhibit the replication of some ssRNA viruses *in vitro*, such as dengue virus [19,94,95], yellow fever virus [96] and Zika virus [97]. It has been proposed that ivermectin can bind to importin α/β1 proteins and reduce the infectivity of coronaviruses. Consequentially, the viral proteins cannot cross the nuclear membrane, resulting in suppression of virus replication [98,99]. Despite positive experimental outcomes, it is essential to thoroughly investigate cases reporting high-grade toxicities, such as neurotoxicity, leakage to the CNS and nerve damage [100–102]. Recently, the concomitant use of ivermectin and HCQ in treating chemoprophylaxis caused by COVID-19 has been reported [103].

### Anti-inflammatory agents

#### Colchicine

Colchicine is a pleiotropic anti-inflammatory cytotoxic agent. One of the proposed pathways is to inhibit the polymerization of microtubules by binding to the tubulin protein. In addition to preventing mitosis, colchicine prevents neutrophil activation and migration to the site of inflammation. This important anti-inflammatory drug is commonly utilized to treat several inflammatory diseases such as gout and inflammatory bowel disease (IBD). Furthermore, several studies have reported efficacy in the treatment of Epstein-Barr virus (EBV) [104], hepatitis virus [105,106], adenoviral and adeno-associated viral vectors [107] and herpes simplex virus type 1 [108]. A cytokine storm leading to hypovolemic shock is a severe outcome of COVID-19. One essential aspect of treating such infectious diseases is to prevent this cytokine storm. In this regard, colchicine can be considered a potential drug for preventing the overproduction of pro-inflammatory cytokines. More specifically, colchicine is a valuable drug for various infectious diseases triggered by over-activation of the IL-1/IL-6 pathway [109]. This drug has been shown to improve other immune-related outcomes in COVID-19 patients, such as acute kidney damage, intravascular coagulation and heart damage [110]. These cases are often related to disease in organ systems largely inhabited by endothelial cells [111,112].

Furthermore, viral infections targeting endothelial cells often induce a significant amount of oxidative stress [113]. Colchicine inhibits the oxidative stress. Cascade can be an important option to improve these complications [114]. Additionally, another factor that aggravates COVID-19 symptoms is the increased abundance of neutrophil cells. As mentioned earlier, colchicine can dampen inflammation by inhibiting the function and migration of these cells [115]. Several laboratories worldwide are currently examining the effect of this drug in improving COVID-19 infection [116]. In the case of colchicine, not all tests are promising. One study found that colchicine at intracellular pH may increase the viral load of SARS-CoV-2 and thus increase inflammation, resulting in more severe symptoms of COVID-19 disease [117].

### Nonsteroidal anti-inflammatory drugs

#### Ibuprofen

Ibuprofen is an NSAID used to suppress inflammation, fever and pain. Research on the effectiveness of this drug on COVID-19 infection is still in the infancy stage of development. Recent studies have shown that the virus must bind with the ACE2 receptors to enter eukaryotic cells [118]. The virus attaches to the cell surface through this receptor. So far, the only logical connection between ibuprofen and the improvement of COVID-19 infection is the inhibitory effect of this drug on the receptor [119]. However, there is a great deal of disagreement among countries about using this drug to improve patient conditions. One of the strongest opponents of using this drug to cure infection caused by COVID-19 is the French scientific community [120]. In France, it is speculated that the use of this drug will increase ACE2 receptors, which will lead to penetration of the virus into cells. After this report, use of this drug in France decreased to 80% [120]. Unlike France, however, the UK noted no evidence of increased vulnerability to COVID-19 infection after taking ibuprofen. However, there are generally some doubts about the association between altered receptor expression (ACE2) at the cellular level and the risk of COVID-19 [121,122]. According to the UK reports, on using ibuprofen or other NSAIDs to treat infection with COVID-19, the risk of infection would be very low because patients usually start taking these drugs after infection, and the ACE2 expression would be increased after the infection stage. In this case, it is difficult to say the drug's direct effect on the observed large number of receptors. In the early phases of the disease, no specific symptoms are detected in most patients. However, after the onset of the storm of cytokines, the symptoms of inflammation intensify suddenly and lead to extensive damage to sensitive tissues such as the lungs. In this case, reducing or even suppressing immune responses can be helpful [123,124]. In summary, although observations and opinions are different, these drugs must be utilized with caution when people are infected with SARS-CoV-2 virus.

#### Naproxen

Naproxen, a member of the NSAID family, reduces fever, pain and inflammation by reducing the mediators involved in inflammation, such as prostaglandins. Given the current crisis caused by the SARS-CoV-2 virus, scientists are finding many ways to fight it. One way is to target the viral N-nucleoprotein to prevent it from binding to the RNA of the virus. This pathway can be an essential strategy to decrease the outbreak of the virus and any possible role associated with the N-nucleoprotein. Previous studies have shown the beneficial effects of naproxen on infections caused by the influenza A virus [125,126]. Today, molecular modeling suggests that the drug binds to N-nucleoprotein. In addition, in laboratory models of SARS-CoV-2–infected cells, naproxen has displayed inhibitory effect on the replication of the viruses in VeroE6 cells [127]. In general, it is suggested that by binding the drug to the viral N-nucleoprotein, the connection between viral RNA and this complex protein would be disrupted, resulting in a reduction in virus replication [5,128]. In general, relying on the lack of scientific evidence of naproxen's severe toxicity and the proven effects of this drug on reducing cytokine storm syndrome (CSS), it can be stated that the use of this drug can reduce the syndromes caused by COVID-19.

### Mucolytic agents

#### Acetylcysteine

This drug is used in the treatment and improvement of some diseases such as cystic fibrosis. Some anti-inflammatory and anti-cytokine activities via the nuclear-factor-kappa-B (NF-κB) pathway have been suggested for this drug [129].

The reason for focusing on this drug to improve the condition of patients with COVID-19 is the effect of acetylcysteine on increasing the production of glutathione (GSH). GSH is an intracellular antioxidant substance that is not able to transport across the membrane. On the other hand, acetylcysteine can easily cross the membrane and enter the cell from the extracellular environment. When acetylcysteine enters the cell, GSH production eventually increases, and the cell's antioxidant power increases during inflammatory diseases [130]. The thickness of the mucous membrane inside the respiratory tract increases significantly in patients with COVID-19. It has been observed that molecules with high disulfide reduction capacity, such as acetylcysteine, can reduce disulfide bonds in the mucosal matrix, thereby reducing the thickness of the mucosal layer. This activity increases with increasing pH of the environment, and the greatest effect is observed at pH levels between 7 and 9 [131]. Also, in some patients with COVID-19, glucose-6-phosphate dehydrogenase (G6PD) deficiency has been shown to accelerate disease progression and intensify the syndromes of the infection. This relationship has led some researchers to consider the use of acetylcysteine in the treatment of COVID-19. By reducing the intracellular level of G6PD, the reductive molecules of glutathione are reduced, and as a result, the reducing agents of disulfide bonds within the mucosal matrix are reduced. These cases ultimately lead to an increase in the thickness of the mucosal layer in patients with COVID-19 and cause shortness of breath. These difficult cases can be cured with the use of acetylcysteine.

On the other hand, the positive effects observed from the consumption of acetylcysteine in patients may also be due to the anti-cytokine activity. These cases need to be further studied. Due to the inherent increase in the level of oxidants in the older body, the risk of death increases in the elderly infected with this virus. Especially in people over the age of 80 and for males, the risk of death is very high. As mentioned, acetylcysteine can delay this process by increasing GSH levels in the cells and can greatly help the recovery of older patients from the disease [132]. These theories are reliable with the detected observations. It has been observed that in patients the effect of normal levels of antioxidants, and serum levels of GSH molecule, is out of balance [133]. On the other hand, as mentioned earlier, ACE2 receptors play a vital role in attaching the virus to the surface of the respiratory cells. Thanks to recent studies, it is known that the amino acid cysteine displays a significant role in the binding of virus S-proteins to these receptors. As a result, the use of acetylcysteine, which ultimately leads to a reduction in disulfide bonds, is a good strategy for treating patients with dehydration [130].

#### Bromhexine hydrochloride

Bromhexine is extracted from vasicine, an alkaloid and a major component of the plant *Justicia* *adhatoda* [134]. Bromhexine, like acetylcysteine, is a drug that helps reduce mucus secretion [135]. Deforming the glycoproteins in the secretions that cause mucosal adhesions [136], it ultimately reduces this property in the mucosal layer and facilitates breathing for patients. It has been prescribed since 1963 as a potent cure for diseases such as asthma [137] and bronchitis [138]. In previous studies, the combined effect of this drug with antibiotics has opened many hopes for the use of this drug in viral infections [139]. Considering the features mentioned for bromhexine, the hypothesis of using this drug to improve patients with COVID-19 has been strengthened.

In addition to the ACE2 membrane surface protein, serine proteinase 2 also plays a significant role in viral attachment to cells. Bromhexine has been shown to be potent in binding to this protein, eventually inhibiting this receptor and preventing the virus from entering human cells. Because the positive effect of this drug in treating MERS and SARS has already been observed [140], many efforts have been made to prove its effectiveness in treating COVID-19 [141]. It is currently used experimentally, about 13% in SARS-CoV-2–infected patients in China. In summary, due to its very low incidence of side effects, the use of bromhexine hydrochloride is recommended to improve infectious disease [138].

### Immunomodulatory drugs

ARDS could happen during COVID-19 infection. This syndrome can cause hyperinflammation and CSS, leading to organ failure [142]. The administration of immunomodulatory drugs could be an effective and promising approach to circumventing these harmful immunological responses [143–145]. Critical immune modulators are discussed below (Table 3).

**Table 3.** Classification of possible COVID-19 treatment in the following categories: immunomodulators, corticosteroids, immunosuppressants and anticoagulants.

Drug	Administration	Mechanism of action	Contraindication	Major side effects	Ref.
Anakinra	5 mg/kg twice a day iv.or 100 mg twice a day sc.	IL-1 receptor antagonist	Hypersensitivity to anakinra and *E. coli* isolated proteins	Injection site reaction, headache	[148]
IFN-1	44 μg sc. (5 doses for 10 days)	Inhibits viral replication, virus maturation and release from infected cells; improves macrophages, T cells and NK cell activity	Hypersensitivity, autoimmune hepatitis, decompensated liver disease	Fever, neutropenia flu-like syndrome, fatigue	[152]
(Methyl)prednisolone	40 mg once or twice daily	Avoids or controls (hyper) inflammation and CSS	Most of the virus vaccines, untreated serious infections	Edema, acne, adrenal suppression	[182]
Dexamethasone	6 mg orally or iv. daily	Anti-inflammatory to inhibit CSS	Hypersensitivity	Edema, hypertension, hyperglycemia	[204]
Hydrocortisone	100 mg by iv. bolus injection followed by 50 mg iv. every 6 h or 200 mg/day by continuous iv. infusion	Anti-inflammatory	Untreated serious infections, hypersensitivity	Adrenal suppression	[173]
Budesonide + formoterol	Continue the administered dose for controlling asthma or COPD	Anti-inflammatory-bronchodilator	Hypersensitivity	Upper respiratory tract infection	[180]
Fingolimod	0.5 mg orally once per day, for 3 days	Sphingosine-1-phosphate receptor regulator	Hypersensitivity, within past 6 months of MI, stroke or angina	Liver enzyme elevations (ALT and AST)	[182]
Leflunomide	50 mg twice daily, three consecutive times, after 20 mg, once daily, for 10 days	DHODH inhibitor, immune regulator	Pregnancy, hypersensitivity, severe liver damage	Elevation of the levels of liver enzyme, ALT and AST	[184]
Thalidomide	100 mg by mouth once daily	Suppresses TNF-α, IL-6	Hypersensitivity, teratogenic drug even in a single dose in pregnant women	Embryo-fetal toxicity	[187,188]
Tocilizumab	400 mg iv. once daily or 8 mg/kg iv. up to 800 mg daily	Monoclonal antibody against IL-6	Hypersensitivity	Increased risk of severe infections (e.g., tuberculosis), hypersensitivity, erythema, pruritis	[192,196]
Sarilumab	400 mg iv. once daily	Monoclonal antibody against IL-6	Hypersensitivity to sarilumab or excipients	Increased risk of severe infections (e.g., tuberculosis), hypersensitivity, erythema, pruritis, elevated liver enzymes (ALT and AST)	[200]
Adalimumab	40 mg sc. every other week	Recombinant human TNF-α immunoglobulin G (IgG)1 monoclonal antibody	-	Upper respiratory tract infection	[205]
Bevacizumab	7.5 mg/kg + 0.9% sodium chloride 100 ml, iv. drip	Recombinant humanized anti VEGF monoclonal antibody	-	Fatigue, nausea	[207]
Ravulizumab	Day 1: (2400 mg for ≥40 to <60 kg, 2700 mg for 60 to100 kg, 3000 mg for ≥100 kg); days 5 and 10: (600 mg for ≥40 to <60 kg or 900 mg for> 60 kg); day 15: (900 mg)	Recombinant monoclonal antibody that inhibits complement pathway	*Neisseria meningitidis* infection and patients who are not vaccinated for *Neisseria* meningitidis	Upper respiratory tract infection	[214]
Lenzilumab	600 mg 1-h iv. infusion every 8 h for 3 doses	Recombinant monoclonal antibody that neutralizes GM-CSF	-	-	[218]
LMW heparin or enoxaparin sodium	40 mg once per day or 40 mg twice per day	Inhibition of factor Xa, antithrombotic agents	Active major bleeding, heparin-induced thrombocytopenia	Hemorrhage	[232,233]
Rivaroxaban	20 mg once daily	Inhibition of factor Xa, platelet activation	Hypersensitivity, active pathological bleeding	Hematoma	[231]

CSS: Cytokine storm syndrome; COPD: Chronic obstructive pulmonary disease; DHODH: Dihydroorotate dehydrogenase; GM-CSF: Granulocyte-macrophage colony-stimulating factor; IFN: Interferon; IL: Interleukin; LMW: Low molecular weight; NK cells: Natural killer cells; VEGF: Vascular endothelial growth factor.

####  Anakinra

Anakinra is an IL-1 receptor antagonist. Anakinra is authorized for use in patients with RA. Moreover, it has also shown therapeutic benefit for severe sepsis in patients with multi-organ dysfunction [146]. An effective strategy of targeting IL-1 has been implemented to eliminate the requirement for mechanical ventilation in COVID-19 patients who are not admitted to ICU [111,147]. Thus, a need for a recombinant human interleukin 1 receptor antagonist (IL-1Ra) (anakinra) is felt. In a retrospective clinical study, anakinra was administered to non-ICU patients with COVID-19; the findings suggested that 5 mg/kg twice a day intravenously is safe and enhanced the clinical outcomes in 72% of patients [148,149]. In another cohort study, anakinra was co-administered in ICU patients who were also placed on mechanical ventilation. Anakinra decreased the requirement for mechanical ventilation and the mortality rate due to ARDS [146].

### *Interferon-alpha & interferon-beta*

IFN-α and interferon-beta (IFN-β) have shown both antiviral and immunomodulatory effects [150]. These classes of IFNs have a direct effect on viral particle replication, protein synthesis and release from infected host cells. Moreover, IFN I enhances macrophage activity [151–153]. As a consequence, IFN I is administered in HBV and HCV [154,155], multiple sclerosis [156] and some types of malignancies such as hairy cell leukemia, melanoma and lymphoma [157–159]. Among the various isoforms of IFN-α and β, IFN-α-2b and IFN-β-1a are the most studied IFNs in COVID-19 [38]. A clinical trial was conducted on patients who were admitted to the Hong Kong hospitals [160]. Their treatments were LPV 400 mg twice a day (b.i.d.)/ritonavir 100 mg b.i.d./ribavirin 400 mg b.i.d./IFN-β-1b 8 million units (3 doses), compared with patients treated without IFN for 14 days. This study indicated that the group receiving combination therapy with IFN had a significantly reduced duration to a negative swab test [160]. Therefore, administration of IFN with other antiviral drugs was effective in earlier viral clearance. In a single-arm prospective clinical trial, patients received IFN-β-1a 44 μg s.c. (5 doses for 10 days) beside HCQ and LPV/ritonavir. This study indicated that administration of IFN also significantly reduced the time of viral clearance and recovery of patients [152].

#### Corticosteroids

There is much controversy in the literature regarding the advantageous effects of corticosteroids in dealing with COVID-19. From the pathophysiological perspective, after SARS-CoV-2 virus enters the host cells, the innate immune system is the initial responder. Unless the inflammatory response is regulated, CSS can occur [161]. CSS induces clotting, hyperinflammation, thromboembolismand ultimately hypovolemic shock [162]. It seems that avoiding these conditions by using corticosteroids is of interest. Still, it should be considered that inflammation is the result of immune activation, and suppressing the entire immune response may cause the relapse of viral replication and reduce viral clearance. Therefore, keeping a balance between immune response and viral replication and infection should be considered (Table 3) [39].

#### Methylprednisolone

Methylprednisolone is an anti-inflammatory drug that interacts with specific cytoplasmic receptors and regulates protein synthesis. Methylprednisolone inhibits the phospholipase A_2_ protein, which plays a major role in synthesizing inflammatory mediators such as prostaglandins and leukotrienes [163]. In an observational study, 40 mg of methylprednisolone administered once or twice daily was evaluated [164]. Results indicated that there was no clinical benefit in the treatment arm receiving methylprednisolone. Methylprednisolone did not influence the duration of hospitalization, virus clearance and duration of symptoms [164].

#### Dexamethasone

Dexamethasone is another corticosteroid with antiemetic properties. Dexamethasone is an agonist for the glucocorticoid receptor (GR) [165]. Its anti-inflammatory effect can control severe allergic symptoms. Therefore, it can be a remedy for asthma, atopic and contact dermatitis, seasonal allergic rhinitis and other disorders related to adrenocortical steroids.

Because this corticosteroid has a prolonged clearance rate, broad-spectrum effects can take place on the innate and adaptive immune systems and can suppress the cytokines' destructive function [166–168]. In a case–control clinical trial for COVID-19, hospitalized patients received 6 mg dexamethasone p.o. or iv. daily for 10 days [169]. Dexamethasone treatment for patients under mechanical ventilation or oxygen demonstrated clinical benefits and an improved mortality rate. However, dexamethasone did not show clinical beneficial effects in patients not using ventilators [169].

#### Hydrocortisone

Hydrocortisone is a corticosteroid that interacts with both glucocorticoid and mineralocorticoid receptors. Some reports indicate that hydrocortisone can inhibit oxidative metabolism, although it has lower anti-inflammatory properties than prednisolone and dexamethasone [170]. Hydrocortisone would be a suitable replacement for those who suffer from cortisol deficiency. It is used to treat a vast range of inflammatory diseases, such as anaphylaxis, angioedema and adrenal crisis. Several methods of administration are used, such as oral, intravenous injection, and topical application [171].

For hospitalized patients suffering from critical conditions such as ARDS and exacerbating conditions, higher doses of corticosteroids are administered. For example, a study suggested that hydrocortisone 50–100 mg as bolus administration and then infusion of 200 mg/day was a suitable regimen for adrenal-insufficient patients with sepsis [172]. Adrenal crisis is a serious cause of death, and once adrenal crisis occurs in COVID-19 patients, the dose of hydrocortisone should be doubled [173].

#### Budesonide/formoterol combination

Budesonide/formoterol combined preparation (Symbicort^®^) is a corticosteroid and beta-2 (β_2_) adrenergic agonist. Budesonide/formoterol is another anti-inflammatory agent and bronchodilator [174]. Inhaled corticosteroids (ICSs) alone or with long-acting β_2_ agonist as a bronchodilator such as budesonide + formoterol was previously used for the management of asthma and chronic obstructive pulmonary disease (COPD) [175,176].

Often, the early signs of COVID-19 are cough and fever. After 8 days, the onset of dyspnea occurs in 20% of patients and is followed by ARDS in hospitalized patients [177]. There is some controversy regarding the use of ICSs in the management of COVID-19 due to some reports of exacerbated effects on viral respiratory infections [178,179]. But it is suggested that patients who have eosinophilic asthma or COPD continue their ICS therapy to reduce symptoms and protect them from viral triggers such as COVID-19 [180].

### Immunosuppressants

#### Fingolimod

Fingolimod is an immunomodulatory drug. It is an analog of sphingosine, which is phosphorylated by sphingosine kinase; it regulates sphingosine 1 phosphate receptor. Fingolimod can influence the aggregation and distribution of lymphocytes. It isolates and captures lymphocytes in the lymph nodes to prevent their contribution to autoimmune diseases [181]. Fingolimod is involved in the treatment of inflammatory diseases of the CNS. Presently, fingolimod is in phase 2 of a clinical trial (NCT04280588) for pneumonia caused by COVID-19. In this nonrandomized trial, fingolimod 0.5 mg was given to patients for 3 days and the change of pneumonia severity on x-ray images was evaluated after 5 days of treatment [182]. Studies are still under way.

#### Leflunomide

Leflunomide is considered for autoimmune disorders, mainly influencing joints. Leflunomide inhibits dihydroorotate dehydrogenase (DHODH) and has a critical role in the synthesis of uridine monophosphate (rUMP), which is vital for DNA and RNA synthesis. This inhibition activates a series of steps that eventually lead to G1-phase cell cycle arrest [183]. It has FDA approval for curing RA and psoriatic arthritis (PsA). Additional clinical studies have been performed on a wide range of other diseases such as Felty's syndrome, Kimura's disease, SLE and Takayasu arteritis.

In one study, leflunomide was evaluated in COVID-19 patients [184]. It was indicated that the patients who received leflunomide had a shorter duration of viral shedding and hospitalization. Moreover, a lower incidence of inflammatory actions and CSS was observed in comparison with those who did not receive leflunomide [184].

#### Thalidomide

Thalidomide is an immunomodulatory drug that inhibits angiogenesis. Nowadays, this drug is prescribed for its anti-inflammatory properties in cancer comorbidities, including multiple myeloma leprosy and graft-versus-host disease. Moreover, thalidomide significantly reduces oxidative stress and TNF-α, IL-1 and IL-6 production [185–187]. One study showed that thalidomide is a good candidate for preventing lung injury during H1N1 influenza infection, as shown by a dampened inflammatory response and prolonged survival in mice [188]. Recently, thalidomide has also been administered in COVID-19 patients for anxiety and nausea. In this way, oxygen consumption is reduced and digestive symptoms are alleviated [189].

### Monoclonal antibodies

As mentioned before, COVID-19 affects several organs, particularly the lungs undergo damage by inflammation and a cytokine storm [111]. Monoclonal antibodies (mABs) would be used to directly bind to the pivotal inflammatory cytokines, prevent an immune response and inhibit organ damage. Some of these mABs are listed in Table 3.

#### Tocilizumab

**Figure 3.**
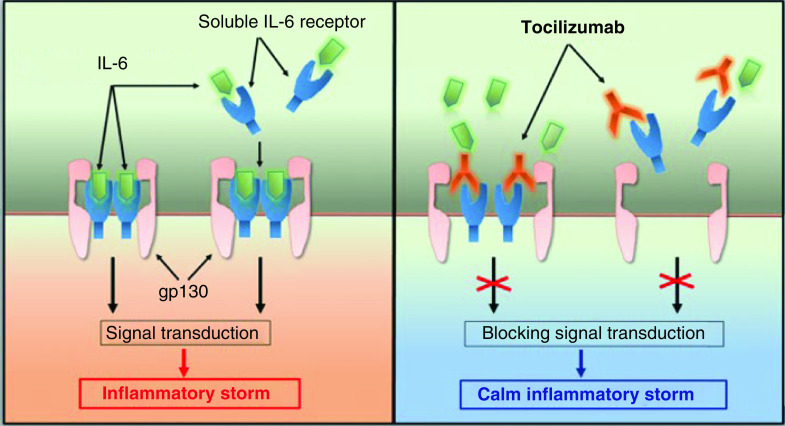
Mechanistic role of tocilizumab in treating COVID-19. Tocilizumab calm inflammatory storm by binding with the receptor of IL-6 (gp130) and reducing its inflammatory properties in the body [195].

This drug is a recombinant humanized mAB IL-6 inhibitor. It binds to the IL-6 receptor and dampens its inflammatory properties [190]. In 2010, the FDA approved the use of tocilizumab for RA and cytokine release syndrome (CRS) caused by chimeric antigen receptor (CAR) T-cells [191]. Some clinical trials on tocilizumab proposed this drug as a promising option in alleviating symptoms in COVID-19 patients [192,193]. COVID-19 patients have higher levels of plasma cytokines,interleukin family (IL-2, IL-6, IL-7, IL-10) and are at high risk for CSS [194]. As depicted in Figure 3, in CRS, IL-6 binds to IL-6R to form a complex that binds to transmembrane glycoprotein 130 (gp130) for subsequent signal transduction and gene expression. The synthesis of acute reactive protein is completed through two signaling pathways. Tocilizumab can prevent CSS by inhibiting active complexes to IL-6Rs [195].

Also, a study on severely ill COVID-19 patients demonstrated that the administration of tocilizumab remarkably reduced fever within a few days and limited the need for oxygen supplementation [196]. However, administration of tocilizumab in COVID-19 patients requires more monitoring and further clinical trials in a larger population to confirm its safety and efficacy [197,198].

#### Sarilumab

Sarilumab is a human monoclonal antibody that is an IL-6 receptor antagonist; it is approved to treat RA in patients with severe or critical COVID-19 [199]. Sarilumab inhibits the binding of IL-6 to its α receptor and is mainly used to treat RA in those who could not tolerate conventional therapies or who have had insufficient feedback to one or more disease-modifying antirheumatic therapeutics [199].

Considering the major role of IL-6 in the pathophysiology of COVID-19, sarilumab as another mAB against IL-6 was suggested to alleviate respiratory symptoms [200]. The level of IL-6 in the serum of patients is excessively increased and results in an exaggerated immune response. So, the administration of sarilumab may be promising in the COVID-19 pandemic. However, careful monitoring of its safety and efficacy, due to some adverse effects such as an indication of severe infections and hypersensitivity, would be considered during and after treatment [201].

#### Adalimumab

Adalimumab is a TNF-α inhibitor that has more advantages than other TNF-α inhibitors due to its large antigen size, which increases the binding affinity to tumor necrosis factor receptor (TNFRS) [202]. TNF-α plays a crucial role in the majority of severe inflammatory mechanisms in the body. It is currently used to treat diseases such as RA, juvenile idiopathic arthritis (JIA), PsA and ankylosing spondylitis (AS).

COVID-19 patients have higher circulating levels of TNF-α in the plasma and tissues [203]. Therefore, the administration of anti-TNF-α, such as adalimumab, would be beneficial for COVID-19 patients. However, anti-TNF-α therapy presents an increased risk for severe infections [204]. In one study, a 30-year-old patient with a history of Crohn's disease was hospitalized with fever and chest pain and tested positive for COVID-19 [205]. He was already under treatment with 3 g mesalazine daily and 40 mg s.c. of adalimumab every other week. Interestingly, his fever and chest pain resolved after 24 hours and he became asymptomatic on the fifth day. It was suggested that the administration of adalimumab might have been beneficial in this case, owing to suppression of the immune system [205].

#### Bevacizumab

Bevacizumab is a humanized mAB that targets VEGF. VEGF is known to be elevated during the hyperinflammation stage and is upregulated in infected respiratory tract epithelial cells [206]. VEGF promotes vasculogenesis and angiogenesis [207] and has an important role in ARDS outcomes [208]. Inhibition of VEGF influences tumors through several mechanisms; it prevents the growth of other vessels and improves the normal function of vessels [209]. Bevacizumab is approved for renal cell carcinoma, colorectal cancer and metastatic breast cancer [207]. Bevacizumab is used in the early stages of treatment when it has not yet been transmitted to other parts of the body. Bevacizumab is not used after surgery. Some trials are currently assessing the safety and efficacy of this mAB in COVID-19 treatment. For example, a randomized, double-blind clinical trial suggested that bevacizumab with other standard care was very effective by enhancing oxygenation and decreasing the duration of oxygen usage [207].

#### Ravulizumab

Ravulizumab is a recombinant mAB that attaches to the complement protein C5 with high affinity and inhibits the production of terminal complement complexes while playing a critical role in the immune system. When C5 is blocked, immune system responsiveness is reduced. Ravulizumab is used to treat adults with paroxysmal nocturnal hemoglobin (PNH) [210]. Recently, ravulizumab was considered as a suitable substitute for eculizumab in PNH patients.

Some reports indicate that in severe COVID-19 cases, some vital molecules of the complement system, such as C3a and C5a, are activated. This complement activation leads to systemic thrombotic microangiopathy (TMA) and multi-organ damage [211,212]. In this circumstance, a complement system inhibitor might be helpful. A clinical trial has been conducted to evaluate the safety and efficacy of ravulizumab in the management of COVID-19 patients with severe pneumonia [213,214].

#### Lenzilumab

Lenzilumab is a recombinant mAB against granulocyte macrophage colony-stimulating factor (GM-CSF). GM-CSF has a critical role as an initiator in inflammatory reactions [215]. Lenzilumab is in Phase III study for COVID-19. Due to the critical role of GM-CSF in pulmonary homeostasis and inflammation, there is a concern to block the receptor of GM-CSF or inhibit GM-CSF signaling in COVID-19 patients [216]. GM-CSF receptor blockade would be advantageous to prevent CSS and inflammatory myeloid cell tissue infiltration induced by COVID-19 [217]. In this regard, a cohort study was conducted to evaluate the safety and efficacy of lenzilumab with a dose of 600 mg iv. for three doses in COVID-19 patients with severe pneumonia. The study suggested that lenzilumab is safe and enhanced the clinical outcomes in hospitalized patients [218].

### Anticoagulants

Systemic inflammatory responses, including the activation of innate immunity by viral infections, lead to activation of coagulation and thrombin pathways called ‘immuno-thrombosis’ [219]. The complement system can activate coagulation factors, and CSS can cause activated vascular endothelial cell damage with prothrombotic characteristics [220,221]. Thus, the use of anticoagulants can reduce the risk of thrombosis in COVID-19 patients (Table 3) [222].

#### Low molecular weight heparin

Low molecular weight heparin (LMWH), or enoxaparin sodium, is an anticoagulant therapy. LMWH activates antithrombin (AT), which in turn inactivates enzymes involved in coagulation. It is used to treat deep vein thrombosis (DVT) and ST-segment elevation myocardial infarction (STEMI) [223].

There are some reports of DVT in hospitalized COVID-19 patients. Although the administration of LMWH would be beneficial for COVID-19 patients with coagulopathy [224–226], identifying the optimal therapeutic window is essential [227]. According to the literature, heparin can also diminish myocardial inflammation and remodeling by decreased collagen deposition *in vivo* [228]. Thus, this cardiac improvement would be advantageous in COVID-19 patients [227]. Moreover, heparin can influence microcirculation and prevent organ damage [229].

#### Rivaroxaban

Rivaroxaban is another anticoagulant. It was the first oral medication that exerts its effects through the direct inhibition of thrombin or factor X_a_. It is a derivative of oxazoline and exerts its pharmacological effect by binding to factor X_a_ [230]. It is used for atrial fibrillation, DVT and pulmonary embolism (PE) [208].

A 79-year-old patient, hospitalized for COVID-19 pneumonia, experienced an episode of acute PE 4 weeks after discharge [231]. However, the patient was receiving rivaroxaban with sufficient drug plasma levels. This case showed concern about rivaroxaban effectiveness in COVID-19. It is recommended that rivaroxaban could be replaced with LMWH until the complete remission of illness [231].

### Cardioprotective agents

#### Aspirin

Aspirin (acetylsalicylic acid [ASA]) is authorized for use in various illnesses such as angina pectoris, colorectal cancer, osteoarthritis and SLE [234]. Aspirin is an inhibitor of cyclooxygenase (COX) 1 (COX-1) and a modifier of COX-2 activity. Unlike other NSAIDs, which reversibly inhibit this enzyme, aspirin binds to COX-2 irreversibly. On the other hand, it irreversibly blocks thromboxane A2 on platelets, preventing platelet aggregation [89].

Aspirin is not ordinarily recommended in the guidelines of COVID-19 treatment. However, it has been confirmed to exhibit antiviral effects by inhibiting prostaglandin E2 (PGE2) in macrophages and upregulate type I IFN, and inhibit virus replication [235]. Under certain circumstances, platelets contribute to innate immune responses. Previous studies indicate that aspirin and antithrombotic drugs could reduce dynamic neutrophil and platelet aggregation in an animal model of lung injury [236]. Among the major complications in patients diagnosed with COVID-19 is the risk for acute lung injury (ALI) and ARDS [237]. Evidence suggests a relationship between taking aspirin prior to hospitalization and ALI/ARDS [238]. A report by Kor *et al.* also showed that 25% of the 3855 patients who received aspirin scored higher on an acute physiologic and chronic health evaluation and had a lower incidence of ALI/ARDS [239]. A study by O'Neal *et al.* suggested that patients who took aspirin and statins before hospitalization had the lowest mortality rates from ALI/ARDS [240].

#### Atorvastatin

**Figure 4.**
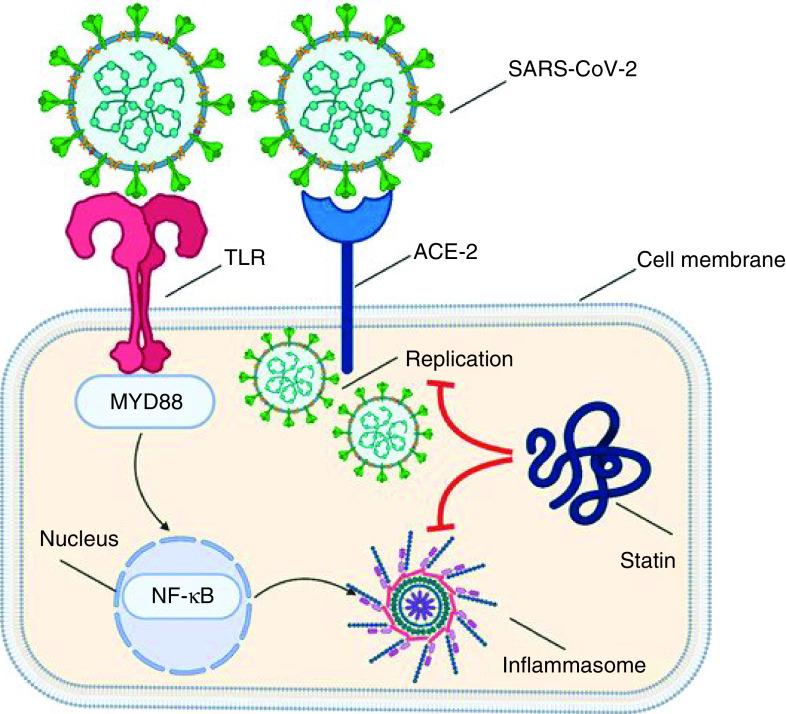
Schematic illustrates statin mechanism of action in the management of COVID-19. Interaction with Toll-like receptors on the host cell membrane increases the expression of the *MYD88* gene and eventually activates NF-κB and triggers the inflammatory cascade. Statins have been shown to stabilize *MYD88* expression levels after a pro-inflammatory trigger and significantly attenuate NF-κB activation [243].

Atorvastatin is an FDA-approved lipid-lowering agent. When combined with dietary modifications, it lowers the risk of cardiovascular events in individuals with cardiac risk factors and abnormal lipid profiles [241]. Atorvastatin hinders β-hydroxy β-methylglutaryl-coenzyme A (HMG-CoA) reductase, the rate-limiting enzyme converting 3-hydroxy-3-methylglutaryl coenzyme A to mevalonate, a precursor of various sterols, including cholesterol [92,242]; furthermore, “*atorvastatin is contraindicated in active liver disease*” [92], unexplained persistent incline of liver transaminase levels, pregnancy and nursing mothers. By interacting with Toll-like receptors on the host cell membrane, COVID-19 increases the expression of the *MYD88* gene and eventually activates NF-κB to activate the inflammatory cascade. Statins have been shown to stabilize MYD88 expression levels after a pro-inflammatory trigger and significantly attenuate NF-κB activation [243]. The schematic mechanism of statins in the management of COVID-19 is shown in Figure 4. Reduced risk of death due to influenza among statin users has been reported [244].

#### Clopidogrel

Clopidogrel is an anticoagulant drug that is used to lessen ‘the rate of stroke in patients with non-ST-segment elevation, acute coronary syndrome’ (ACS), acute ‘ST-elevation MI, and unstable angina/non-ST-elevation MI’ [245]. Clopidogrel is sometimes administered in combination with ASA [246]. Clopidogrel is a prodrug that is absorbed in the intestine and is converted to thiol-containing active metabolites that are able to inhibit platelet aggregation. The active metabolites of clopidogrel irreversibly and selectively hinder the ‘binding of adenosine diphosphate (ADP) to P2Y12 receptor’ on platelets and ‘ADP mediated activation of the glycoprotein IIb/IIIa complex’, thus inhibiting the aggregation of platelets [247]. Several agents used in the treatment of COVID-19 interact with oral antiplatelet agents. It has been reported that LPV/ritonavir, a protease inhibitor that hinders cytochrome P450 3A4 (CYP3A4) metabolism, may lead to decreased serum concentration and affect clopidogrel pharmacokinetic properties [248,249].

Patients with COVID-19 may experience hypoxia, endothelial dysfunction and alveolar involvement, which in turn cause micro-pulmonary thrombosis. The effect of antiplatelet therapy on arterial oxygenation in patients was assessed in a clinical setting [250]. Antiplatelet therapy is effective in improving the partial pressure of arterial oxygen (mmHg) PaO_2_/fractional inspired oxygen (FiO_2_) ratio in patients by preventing the formation of clots in the capillaries of the lung and improving the function of megakaryocytes and platelet adhesion. Inhibition and modulation of COX by clopidogrel in platelets as well as megakaryocytes can prevent the formation of thrombosis and increase the level of PaO_2_ [250].

### Tyrosine kinase inhibitors

#### Imatinib

Imatinib is a bcr-abl tyrosine kinase inhibitor. Imatinib also inhibits tyrosine kinase receptors involved in the function of stem cell factor (SCF), platelet-derived growth factor (PDGF) and c-kit and in turn inhibits PDGF- and SCF-mediated cellular action [251].

Imatinib is a curative therapy prescribed to adolescent patients with chronic myeloid leukemia ‘Philadelphia chromosome-positive’ in blast crisis, in accelerated phase, and in chronic phase following failure of IFN-α treatment [252]. It is also used in patients with recurrent disease after stem cell transplantation and individuals who do not respond to IFN treatment [253]. Besides the conventional mechanism of action, imatinib also exhibits immunomodulatory properties that assist in COVID-19 management. Considering hyperinflammation in COVID-19, the role of imatinib in modulating host immune response is noteworthy. Imatinib exhibits antiviral properties *in vitro* against two viruses phylogenetically related to SARS-CoV-2, SARS and MERS. In this sense, Abelson kinases type 2 (ABL2) seem to be involved in the virus–cell fusion process. Inhibition of ABL2 could control viral entry into the host cells. Imatinib also inhibits c-KIT and PDGF receptors. Imatinib has been shown to prevent lung injury in animal models of COVID-19 by reducing pulmonary edema, it prevents histological damage and it improves endothelial barrier dysfunction. This phenomenon is likely mediated by reducing pro-inflammatory cytokine release such as IL-6 and TNF-α. These effects could be implemented by inhibiting transcription factor NF-κB based on recent *in vivo* and *in vitro* evidence [254,255].

#### Acalabrutinib

Acalabrutinib is the inhibitor of Bruton tyrosine kinase (BTK). Acalabrutinib – and its active metabolite, ACP-5862 –bind to the active site of BTK, leading to the inhibition of BTK. BTK signaling triggers B-cell proliferation [256]. This drug could also inhibit the activation of downstream signaling molecules of BTK, such as CD86 and CD69, impairing the expansion and survival of malignant B-cells [257].

Acalabrutinib is used to treat patients who are diagnosed with mantle cell lymphoma. Serious and opportunistic infections, hemorrhage and cytopenia are among the significant side effects of this drug [258]. A study on patients who were treated with acalabrutinib for 10–14 days revealed that the levels of IL-6 in serum decreased following treatment. By the end of the treatment, 73% and 25% of patients that were on supplemental oxygen or mechanical ventilation, respectively, were discharged on room air [259]. Considering the role of acalabrutinib in COVID-19 treatment, when a macrophage ingests a viral particle, it triggers a signaling pathway that proceeds through BTK [260]. Treatment with BTK inhibitors in patients with leukemia has been very effective and shows a good safety profile, even among patients who are on treatment for years. The hypothesis is that by inhibiting BTK, the inflammatory balance within the lungs is improved and CSS is prevented, and ultimately patients can breathe better. Although this complication is uncommon, BTK inhibitors, such as acalabrutinib, might help control macrophage-dominated inflammatory responses.

Blood samples collected from COVID-19 patients showed that BTK was active in all [261]. In a clinical study on a small group of hospitalized patients, acalabrutinib was administered. Within 10–14 days of therapy, their oxygen supply and inflammatory factors, such as CRP and IL-6, improved significantly [260]. The effects of acalabrutinib are evident in patients receiving supplemental oxygen, such as mechanical ventilation. Half of the patients who used the mechanical ventilation died after receiving acalabrutinib. In this study, no side effects were observed in the long-term use of acalabrutinib, advocating its beneficial effects in COVID-19 [260].

#### Fedratinib

Fedratinib is a tyrosine kinase inhibitor acting on Janus-associated kinase 2 (JAK2) and FMS-like tyrosine kinase 3 (FLT3) [262]. Fedratinib is indicated in the management of patients with myeloproliferative diseases [263]. Fedratinib suppresses the production of several cytokines, preventing CSS in COVID-19 patients. Fedratinib is also prescribed in combination with other antiviral drugs [264].

### Serotherapy

#### Convalescent plasma transfusion

Since the 20th century, convalescent plasma transfusion has been recognized as an alternative therapeutic plan of action in preventing and managing several infectious diseases and is thought to induce passive immunization. Convalescent plasma is drawn from a patient who has recently survived an infectious disease and whose plasma is rich in antibodies that can help another person who is suffering from the same infectious disease. It has been reported that early administration of convalescent plasma therapy remarkably lowered the mortality rate in respiratory viral infectious diseases during previous pandemics, including HINI influenza, SARS and MERS [265]. Convalescent plasma contains defensins, clotting factors, anti-inflammatory cytokines, neutralizing antibodies and other proteins that can induce immunomodulatory responses and avoid the CSS and ARDS complications common in SARS-CoV-2 infection. This therapeutic approach is safe and does not exhibit significant drug interaction complications, especially in patients with underlying diseases [4,265]. Apheresis is the recommended method of plasma acquisition from recovered donors. Pre-donation assessments, such as ABO-compatibility, are essential to prevent unwanted reactions. Neutralizing antibodies in convalescent plasma are beneficial in SARS-CoV-2 clearance and induction of passive immunity. Also, non-neutralizing antibodies in convalescent plasma, such as IgM and IgG, could induce protective and/or therapeutic effects against COVID-19. Therefore, convalescent plasma transfusion would be considered a safe therapeutic or prophylactic agent against SARS-CoV-2 [265]. Although the volume of plasma transfusion and neutralizing antibody titers are variable, there is no correlation between these parameters and clinical response [266]. The recommended amount of convalescent plasma transfusion proposed is 200 (ml) with a 1:640 ratio neutralizing antibody titers. Still, more clinical trials should be implemented to validate these values. Outcomes of a recent study indicated that convalescent plasma transfusion in severe cases (COVID-19) was significantly linked with improving oxyhemoglobin saturation within 3 days of hospitalization. Also, lymphocyte count was significantly enhanced, and CRP was reduced after convalescent plasma transfusion. This study on plasma transfusion in severe cases of COVID-19 reported no significant adverse reaction related to convalescent plasma transfusion. A recent randomized clinical trial study on hospitalized patients conducted in the Netherlands compared the efficacy of convalescent plasma transfusion (300 ml with neutralizing antibody titers of at least 1:80 ratio) with the standard of care therapy [267]. This study revealed no statistically significant difference in regard to mortality rate, disease severity and length of hospital stay between those who received convalescent plasma and the control arm that received standard therapy [267]. The results of a case series study on the efficacy of convalescent plasma transfusion in a hospitalized Swedish population who were severely ill due to COVID-19 infection revealed that although laboratory and inspiratory data, such as CRP and PaO_2_/FiO_2_ ratio, were improved in the convalescent plasma group, the clinical status was the same in both treatment groups [268]. However, this study suffers from a small sample size and short-term patient follow-up. The results of this study emphasized the safety of a convalescent plasma infusion, with no adverse reactions after ABO-compatibility confirmation [268]. The incidence of venous thromboembolism was high in both case and control groups, but it was not higher in the convalescent plasma therapy group [268]. Finally, in August 2020, the FDA confirmed emergency use authorization (EUA) of convalescent plasma therapy to manage critically ill COVID-19 hospitalized patients. The FDA emphasized that plasma should be obtained from COVID-19 recovered patients who did not have clinically relevant symptoms for the last 10 days and who had laboratory-confirmed negative SARS-CoV-2 tests. Plasma has FDA approval for a ‘serious or immediately life-threatening COVID-19’ infection [269].

#### Intravenous immunoglobulin

Since the most effective therapeutic agents in COVID-19 management have emerged from the immunomodulation basis, the administration of intravenous immunoglobulin (IVIG) would be a promising strategy. IVIG is a liquid human immunoglobulin that contains polyclonal IgG antibodies [270]. IVIG had been introduced as an efficient agent in many viral and bacterial infections, inflammatory diseases and autoimmune diseases. IVIG could act as an immunomodulator through the inhibition of pro-inflammatory cytokines. IVIG immediately enhances blood IgG levels that can neutralize SARS-CoV-2 exogenous antigens. Also, it can enhance circulating lymphocytes and innate immune cells. Because there is a lack of selective antibodies in the treatment of the new SARS-CoV-2, the efficacy of IVIG administration in COVID-19 is still controversial [270]. It has been hypothesized that IVIG administration in combination with dexamethasone and IFN-ß could be beneficial for COVID-19 patients who progressed to ARDS [271]. A report of a multi-center retrospective cohort study on assessment of IVIG efficacy in COVID-19 management revealed that early administration (<7 days of hospital admission) of a high dose of IVIG (>15 g/day) resulted in reduced 60-day fatality in critically ill patients [270]. A serial case study on patients showed that administration of high-dose IVIG (with a dosage of 0.3–0.5 g/kg/day for 5 days) in the early stages of COVID-19 disease was significantly associated with the blockage of disease progression and improvement in clinical outcome [272]. It has been reported that high-dose IVIG would be a promising immunomodulatory therapy in prophylaxis and management of inflammatory and infectious diseases, particularly in immune-compromised patients. However, further larger, randomized, controlled trials are needed to support the efficacy and safety of IVIG therapy at different stages of COVID-19 disease.

### Other treatments

#### Vitamin C

Ascorbic acid, also known as vitamin C, is an antioxidant agent that is a potential scavenger of the harmful reactive oxygen species (ROS) and prevents oxidative stress damage to cells and tissues. Vitamin C also plays a role as an essential supporter of a healthy immune system [273]. Vitamin C can inhibit NF-κB, a crucial factor in immune defense activity, by regulating the production of cytokines, chemokines and inflammatory mediators [274,275]. Also, the action of vitamin C in the inhibition of IL-6 and TNF-α is significant in terms of inflammatory and infectious disease management. Vitamin C can regulate the production of T lymphocytes, B lymphocytes and natural killer NK cells and can decrease the signaling responses of GM-CSF. According to the aforementioned mechanisms, vitamin C could be a promising agent in managing CSS [275,276]. During viral or bacterial infections, the level of vitamin C is diminished, and the amount of this reduction is correlated with the severity of the infectious disease. A high-dose intravenous injection of vitamin C may be beneficial in severe infections. Also, there are some suggestion of the direct viricidal effect of vitamin C [276]. A recent study indicated that a high intravenous dose of vitamin C (15 g/day for 4 days) in patients with severe cases of COVID-19 who progressed to ARDS could reduce the mortality rate [274]. Since oxidative stress has a crucial role in the pathophysiology of COVID-19, vitamin C administration, as an antioxidant agent, can prevent oxidative damage to the bronchial epithelium and further ROS-induced lung damage. It could also be beneficial in the management of COVID-19-induced ARDS and respiratory failure. A recently published meta-analysis showed that high-dose intravenous administration of vitamin C could reduce the length of hospitalization, the duration of mechanical ventilation requirements [276] and ICU mortality rates [277]. Also, it has been reported that high-dose intravenous administration of vitamin C (200 mg/kg) could reduce the incidence and score of multi-organ failure (MOF) in critically ill COVID-19 patients with sepsis or ARDS. Some reports claim that high-dose intravenous vitamin C administration (10–20 g/day infusion over 8–10 hours) in the Chinese population with COVID-19 could enhance the oxygenation index [277]. A multi-center, randomized clinical trial on COVID-19 patients evaluating the safety and efficacy of high-dose intravenous vitamin C administration started in February 2020. Results of this study would be helpful to support the efficacy and safety [275]. Since vitamin C is a safe and inexpensive nutritional agent, consideration of vitamin C administration during the COVID-19 pandemic would be promising [276]. Administration of intravenous vitamin C with doses of up to 1.5 g/kg/day would be considered safe with no severe adverse drug reactions (ADRs) [277].

#### Deferoxamine

It is suggested that SARS-CoV-2 can damage hemoglobin molecules. New SARS-CoV-2 can damage the beta chain of hemoglobin, resulting in the separation of iron from the heme molecule. Hence, iron overload would be a common consequence of SARS-CoV-2 infection. Recent reports show many COVID-19 patients had high serum ferritin values [278]. The released iron can be converted to porphyrin, which might progress to hypoxemia. Due to disturbance in oxygen delivery, MOF would be expected in these COVID-19 patients [273]. Iron overload and iron toxicity have multi-stage consequences. The first stage is GI events such as nausea/vomiting and diarrhea, the second stage is the apparent recovery of the first-stage symptoms and the third stage is metabolic acidosis, septic shock, ARDS and MOF. The latest stage of iron overload would be hepatic failure and elevation in the levels of aminotransferase. Since these symptoms are common in severe COVID-19 cases, the management of iron overload using an iron chelating agent, such as deferoxamine, would be beneficial in these patients. It has been suggested that iron level assessments in these patients would be helpful in the initiation of deferoxamine therapy. If the serum iron level passes over 500 μg/dl in hospitalized COVID-19 patients, deferoxamine can be prescribed, with a loading dose of 1000 mg and followed by 500 mg q4h for two other doses. In some patients, doses might be enhanced up to 6 g/day [279]. Also, some evidence shows that the iron overload process could enhance viral replication and disease severity. It has been suggested that iron chelating agents could be beneficial in preventing lung injury and lung fibrosis in patients through the reduction of viral replication. Also, it has been suggested that deferoxamine could have synergistic effects with antiviral agents, such as remdesivir, in reducing the viral replication cycle.

Another possible mechanism of deferoxamine in decreasing the COVID-19 severity course would be through reducing IL-6 values and diminished endothelial inflammation, which could prevent COVID-19-induced MOF [278]. Hyperferritinemic syndrome has clinical, laboratory and atopic similarities to COVID-19 pneumonia, including lymphopenia, coagulopathy, enhanced transaminases, septic shock, CSS and MOF. It could be concluded that COVID-19 systemic inflammation would be a part of hyperferritinemia syndrome [280,281]. It has been hypothesized that deferoxamine would be helpful in the management of COVID-19 due to its *in vitro* and *in vivo* immunomodulatory and antiviral effects. Also, deferoxamine can reduce viral replication through the induction of the iron deprivation that is a crucial element in the SARS-CoV-2 replication process [282,283]. So, iron chelating agents such as deferoxamine would be notable supportive therapy in the treatment of critically ill COVID-19 patients. However, randomized, controlled trials are required to authorize the risks and benefits of deferoxamine administration in the management of COVID-19 patients.

#### Metformin

Metformin, a biguanide antidiabetic agent, has been recently introduced as a promising therapeutic agent in COVID-19 pneumonia patients with underlying Type 2 diabetes mellitus (DM) due to its pleiotropic effects. The possible mechanisms of metformin in COVID-19 management were subjected to control blood sugar levels, body weight reduction, diminished insulin resistance and prevent hyperactivation immune responses. ARDS is likely prevented through inhibition of the mammalian target of rapamycin pathway and neutrophil reduction. Additionally, the blockade of mitochondrial ROS signaling pathway can occur through cellular pH enhancement via endosomal Na^+^/H^+^ exchangers and interference with viral endocytosis [284]. Viral penetration inhibition through the activation of mitogen-activated protein kinase (AMPK) and phosphorylation of ACE2 at Ser680 residue in human umbilical vein endothelial cells has been suggested. This phosphorylation process can induce steric hindrance and prevent the binding of ACE2 to the SARS-CoV-2 receptor-binding domain [285,286]. ACE2, a peptide with anti-inflammatory properties, is considered as a protective marker in the renin-angiotensin-aldosterone system (RAAS) with several beneficial cardiopulmonary effects [287–289]. Also, it has been hypothesized that metformin can reverse COVID-19-induced lung fibrosis [284]. Results of some observational and retrospective studies revealed that metformin administration in DM patients hospitalized due to COVID-19 infection decreased fatality rates [290–292]. Although DM has been considered an important risk factor for COVID-19, it seems that in COVID-19 cases with DM who were receiving metformin as an antidiabetic agent, minor lung injury and ARDS have occurred due to the protective role of metformin in AMPK activation and ACE2 phosphorylation, which results in an ACE2 extended half-life and higher circulating ACE2 levels [288]. So, it seems that metformin would be a potential therapeutic agent in patients in acute chronic or recovery stages of COVID-19, but more extensive, controlled trials are needed to prove it is efficacious in COVID-19 management among both diabetic and nondiabetic patients. Finally, it has been warned that the administration of metformin in COVID-19 patients should be discontinued promptly if severe GI effects, hypoxia, lactic acidosis of MOF occur [287].

### Mesenchymal stem cells

Mesenchymal stem cells (MSCs) are multipotent cells that would be considered in many inflammatory and immunologic diseases. The most common sources of MSC transplantation are the umbilical cord, bone marrow and adipose tissue. The possible mechanism of MSC therapy in COVID-19 management is by initiating MSC differentiation [293]. Also, MSC therapy could induce tissue repair through various mechanisms, including secretion of anti-inflammatory cytokines, angiogenesis induction, immunomodulatory potential and extracellular vesicle release. MSC could induce its innate and adaptive immunomodulatory capacities through the secretion of soluble factors, such as nitric oxide (NO), TGF-ß1 and PGE2, that can inhibit the maturation of dendritic cells and block the function of lymphocytes such as T-cells, B-cells and NK cells and ameliorate symptoms of COVID-19-induced ARDS [293]. It has been hypothesized that dsRNA of SARS-CoV-2 could induce the immunomodulatory effects of MSC through the enhancement of Toll-like receptor on MSC. It has also been reported that perfusion of 1 × 10^6^ cells/kg through MSC transplantation could ameliorate all clinical symptoms related to COVID-19 pneumonia [294]. Recent studies evaluating the MSC efficacy in Chinese patients revealed that MSC therapy (infusion of 7 × 10^6^ cells/kg allogeneic human umbilical cord MSC) could significantly induce symptom reversal with no severe adverse reactions [295,296]. Since MSCs could express low levels of human leukocyte antigens, they can be used as allogenic transplantation to manage acute inflammatory disorders. They are capable of relieving lung injury about 30 minutes after intravenous perfusion by preventing accumulation in the pulmonary vascular area. MSCs are capable of directly transferring mitochondrial cells to respiratory alveolar epithelial cells. They also have the potential to repair ARDS-induced alveolar capillary wall damage via the secretion of FGF7 and Ang1. An important advantage of MSC therapy is its potential to promote and suppress inflammatory responses based on the environmental situation. Hence, MSC therapy could be an option in the management of severe COVID-19 cases that progress to ARDS, septic shock, CSS and MOF, especially in heart, kidney and lung injury cases [293]. A study from China revealed that MSC therapy in patients with severe infection (COVID-19) was accompanied by a decrease in immune system overactivation and a decrease in lung repair through the induction of the lung microenvironment process [297]. A recent study on assessment of MSC therapy's efficacy and safety in COVID-19 management revealed that inflammatory indices such as WBC, pro-calcitonin, CRP and IL-6 were unchanged after MSC perfusion; IgG and IgM were also unchanged after MSC therapy. Still, lactate, cardiac troponin T and creatinine kinase MB were significantly increased [298]. According to this study, all severe cases recovered after MSC therapy. Only three cases experienced therapy-related adverse reactions, including hepatic dysfunction, skin rash and heart failure. So, MSC therapy would be considered an alternative therapy for critically ill COVID-19 patients who progressed to ARDS. Caution is required in cases with metabolic acidosis and coronary heart disease (CHD). Furthermore, the infusion rate should be slow enough to avoid heart failure incidence during MSC perfusion [298]. A summary of possible COVID-19 treatment is given in Table 4.

**Table 4.** Classification of possible COVID-19 treatment mentioned in the “other treatment” section.

Drug	Administration	Mechanism of action	Contraindications	Major side effects	Ref.
Convalescent plasma transfusion	iv.: the optimal dose is unknown but the recommended dose is 1–2 units (≈200–250 ml/unit)	Neutralizing antibodies in convalescent plasma are effective in SARS-CoV-2 clearance and induction of passive immunity. Also, non-neutralizing antibodies in convalescent plasma, such as IgM and IgG, could induce protective and/or therapeutic effects against COVID-19	No significant contraindication	Transfusion reactions, including allergic reaction, anaphylactic reaction, transfusion-related ALI, transfusion-associated circulatory overload, hemolysis, interference with SARS-CoV-2 vaccination, reduction in efficacy of vaccination and antibody-dependent enhancement	[238,265]
IVIG	iv.; 0.3–0.5 g/kg/day for 3–5 days	IVIG could act as an immunomodulator through the inhibition of pro-inflammatory cytokines. IVIG immediately enhances blood IgG levels that can neutralize SARS-CoV-2 exogenous antigens. Also, it can regulate immune media by enhancing the capacity of natural immune cells and lymphocytes	No significant contraindication	Anaphylaxis reactions, TRALI, thromboembolic events, acute kidney injury, hemolysis and hyponatremia	[238,270,272]
Vitamin C	iv.; 10–20 g/day infusion over 8–10 h	Inhibition of IL-6 and TNF-α and regulation of the production of T-cells, B-cells, and NK cells and reduction of the signaling responses of GM-CSF. Also, there are some suggestions on the direct viricidal effect of vitamin C	No significant contraindication	Nephropathy and nephrolithiasis	[274–276]
Deferoxamine	iv.; a loading dose of 1000 mg and then 500 mg q4h for two other doses	An antidote of excess iron that could show synergistic effects with antiviral agents such as remdesivir in reducing the viral replication cycle	Hypersensitivity to deferoxamine, severe renal disease and anuria	ARDS, infusion reactions, and acute kidney injury	[278,279]
MSC	iv.	MSC could stimulate the differentiation process and have immunomodulatory effects and induce tissue repair. MSC could induce its immunomodulatory capacities through secretion of soluble factors, including NO, TGF-ß1, PGE2, and so on, that can block the maturation of dendritic cells	No significant contraindication	No significant adverse reactions	[293,295]
Metformin	Oral	Metformin could diminish insulin resistance, prevent immune hyperactivation and ARDS occurrence through the inhibition of the mTOR pathway. Furthermore, it could show anti-inflammatory effects and neutrophil reduction, blockade of mitochondrial ROS signaling pathway, cellular pH enhancement through endosomal Na+/H+ exchangers.The interference with viral endocytosis and viral penetration inhibition through the activation of MAPK and phosphorylation of ACE2 are among other metformin actions	Severe renal failure (GFR <30); acute or chronic metabolic acidosis with or without coma, including DKA; and severe hepatic failure	GI side effects including diarrhea, nausea, and vomiting; flushing; chest pain; palpitations; and lactic acidosis	[284–286]

ALI: Acute lung injury; ARDS: Acute respiratory distress syndrome; ACE2: Angiotensin-converting enzyme 2; GFR: Glomerular filtration rate; is a test used to check how well the kidneys are working, IgG: Immunoglobulin G; IgM: Immunoglobulin M; IVIG: Intravenous immunoglobulin; MAPK: Mitogen-activated protein kinase; mTOR: Mammalian target of rapamycin; MSC: Mesenchymal stem cells; NO: Nitric oxide, PGE2: Prostaglandin E2; ROS: Reactive oxygen species.

## Latest recommendations on potential COVID-19 treatment options

Although numerous drugs and clinical trials have been reviewed in this paper, due to this pandemic's economic, social and health concerns, an appropriate selection of therapeutic regimens according to a patient-specific status is essential. So, early diagnosis, close patient monitoring and the selection of appropriate therapeutic options based on disease severity should be scheduled. In this regard, a list of recommended therapeutic regimens for COVID-19 management based on the severity of the infection and stage of the disease is given in Table 5 according to the latest version of the NIH COVID-19 treatment guidelines [299].

**Table 5.** A summary of the latest recommendations on potential COVID-19 treatment options.

Therapeutic options	Latest status	Comments
Remdesivir	FDA has recommended the use	FDA approved for the management of hospitalized COVID-19 patients (elderly and pediatrics with an age ≥12 years old and a weight ≥40 kg)
CQ or HCQ ± AZN	Recommended against the use	
Ivermectin	Recommended against its use	Only use in the context of clinical trials
LPV/ritonavir and other HIV protease inhibitors	Recommended against its use	Only use in the context of clinical trials
Convalescent plasma	Insufficient data available in support of its use or against the use	
Ig: SARS-CoV-2 specific	Inadequate data available in support of SARS-CoV-2 Ig use or against the use	
Ig: non-SARS-CoV-2 specific	Recommended against the use	Only use in the context of clinical trials; administration of IVIG in management of complications that may occur during COVID-19 infection course should not be precluded
MSCs	Recommended against the use	Only use in the context of clinical trials
Corticosteroids	Use of dexamethasone (other glucocorticoids) ± remdesivir recommended	Indicated for cases with severe COVID-19 who may also exhibit SIRS, ARDS, multi-organ dysfunction and lung injury
Anakinra	Inadequate data available to either suggest the use or recommend against the use of anakinra	
IFN-β	Inadequate data available to either suggest the use or recommend against the use of IFN-β	Only use in mild and moderate COVID-19 infection and <7 days from onset of signs and symptoms in the context of clinical trials
Anti-IL-6 mAB (siltuximab) and anti-IL-6 receptor monoclonal antibodies (tocilizumab and sarilumab)	Recommended against the use	According to the REMAP-CAP trial, a single dose of tocilizumab (8 mg/kg; max: 800 mg) might be considered as an adjunctive therapy in combination with dexamethasone in patients who are within first 24 h ICU admission and require mechanical ventilation and patients who experience rapid progression to respiratory failure
IFN-α or β	Recommended against the use in severely and critically ill COVID-19 patients	Only used in the context of clinical trials
BTK inhibitors (acalabrutinib, ibrutinib, zanubrutinib)	Use not recommended	Only use in the context of clinical trials
Antithrombotic therapy	Consider therapy based on patient's status	In outpatient setting of COVID-19 management, anticoagulants/antiplatelet treatment should not be started for the prevention of thrombotic events. For hospitalized COVID-19 patients, prophylactic anticoagulation should be considered unless contraindications recorded for the patient
Vitamin C	Insufficient data available	
Vitamin D	Insufficient data available	
Zinc supplementation	Insufficient data available	

AZN: Azithromycin; ARDS: Acute respiratory distress syndrome; BTK: Bruton tyrosine kinase; CQ: Chloroquine; HCQ: Hydroxychloroquine; Ig: Immunoglobulin; IVIG: Intravenous immunoglobulin; LPV: Lonapiravir; mAB: monoclonal antibody, MSCs: Mesenchymal stem cells; REMAP-CAP: A randomized, embedded, multi-factorial, adaptive platform trial for community-acquired pneumonia; SIRS: Systemic inflammatory response syndrome.

**Figure 5.**
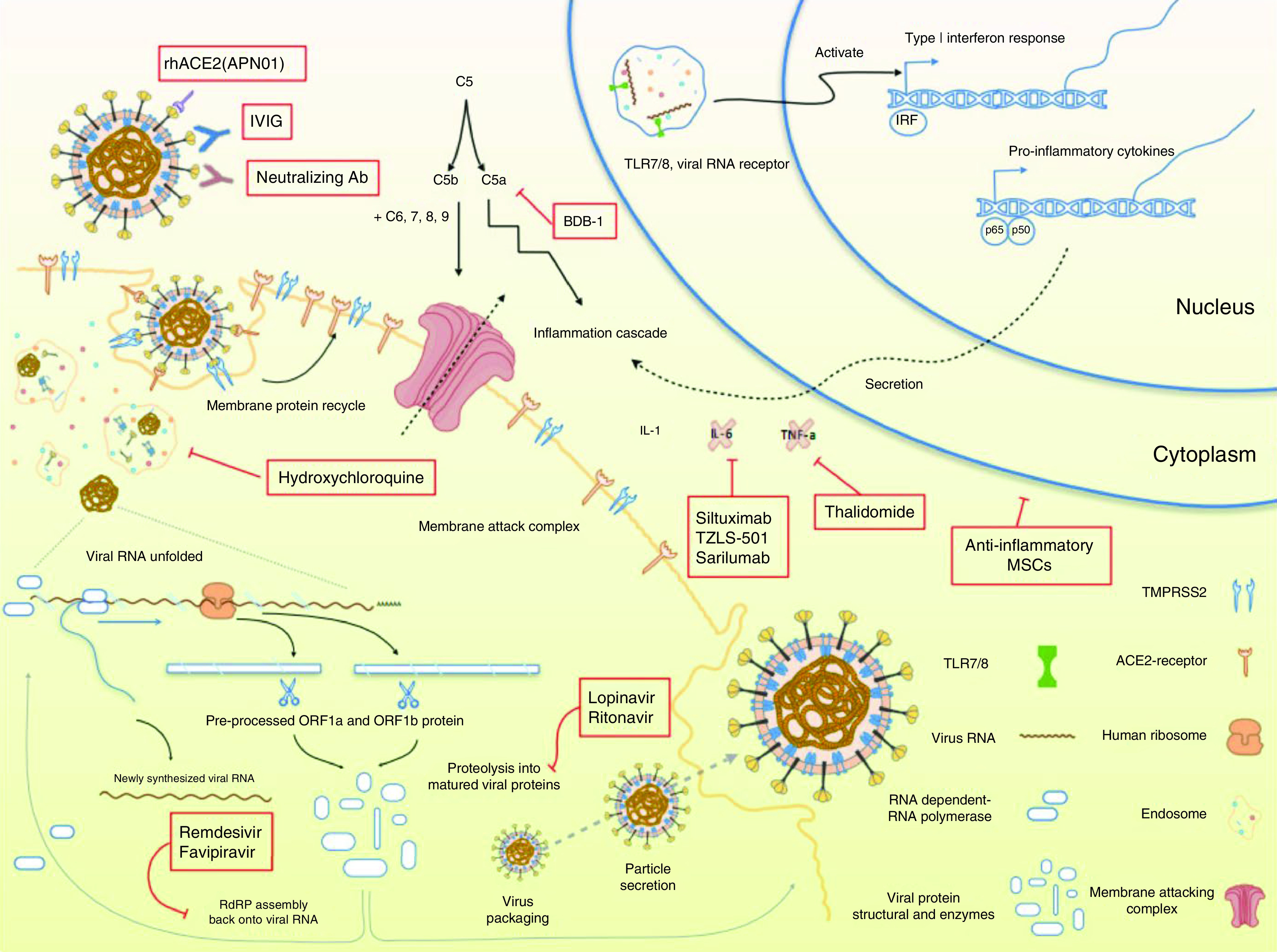
Schematic illustration of different types of SARS-CoV-2 treatments and mechanisms of action. The various repurposed drugs and novel therapeutic approaches undergoing clinical trial against COVID-19 in the context of host pathways and virus replication mechanisms are summarized [300]. Hydroxychloroquine prevents the virus from entering the human body. Thalidomide reduces oxidative stress and TNF-α, IL-1 and IL-6 production. Anti-IL-6 monoclonal antibody (siltuximab) and anti-IL-6 receptor monoclonal antibody (sarilumab) reduce cytokine storm syndrome (CSS). Intravenous immunoglobulin (IVIG) immediately enhances blood immunoglobulin G (IgG) levels that can neutralize SARS-CoV-2 exogenous antigens. IFN-α and IFN-β can directly affect virus particle replication and viral protein synthesis and can diminish virus release from infected host cells. Antiviral drugs (remdesivir, favipiravir, lopinavir/ritonavir) prevent transcription of the RNA virus [300].

An overview of existing drugs in the treatment of COVID-19 is illustrated in Figure 5. HQ and bromhexine prevent the virus from entering the human body. Corticosteroids inhibit CSS, and thalidomide reduces oxidative stress and TNF-α, IL-1 and IL-6 production. Anti-IL-6 monoclonal antibody (siltuximab) and anti-IL-6 receptor monoclonal antibodies (tocilizumab and sarilumab) reduce CSS. IVIG immediately enhances blood IgG levels that can neutralize SARS-CoV-2 exogenous antigens. IFN-α and IFN-β can directly affect virus particle replication and viral protein synthesis and can diminish virus release from infected host cells. Antiviral drugs (remdesivir, favipiravir, lopinavir/ritonavir) prevent transcription of the RNA virus.

## Conclusion

The new COVID-19 rapidly spread around the world, and because of a high transmission rate, it became a pandemic within a few months. Many people suffer from this disease, and over 5 million deaths have been recorded. COVID-19 created one of the greatest global economic crises in history. The economic crises and control measures negatively impacted businesses and working conditions and highlighted inequalities in several communities. Although the first COVID-19 vaccine was administered in December 2020, immunization of 7.8 billion people worldwide is challenging due to the limited supply of vaccines and the new strains of the virus. In order to control and mitigate COVID-19, as well as address the immediate and long-term health outcomes, it is essential to have comprehensive information about the nature of COVID-19: its evolution, characteristics, mode of action and transmission. Due to the circulation of highly transmissible variants, more infections are likely, and unvaccinated people are most at risk. No vaccine is 100% effective; a small percentage of vaccinated people can also become infected, and some breakthrough infections are to be expected. As the delta variant of the coronavirus spreads globally, it is important to develop efficient therapeutic strategies to protect infected patients against severe illness, hospitalization and death. To better understand the risk factors related to epidemic-prone diseases such as COVID-19, an in-depth understanding of the ecological and genetic risk factors is required. One of the best ways to mitigate and prevent transmission of emerging epidemic-prone diseases such as COVID-19 is to understand the virus and how it spreads early on to enable quick intervention strategies. Concerted efforts among communities are vital for this effort. To sum up, regarding the vast spread of COVID-19 worldwide, efficacious medical treatment against COVID-19 should remain a priority to control the growing epidemic and treat COVID-19 patients.

## Future perspective

The COVID-19 pandemic has proven to be an unprecedented catastrophe affecting all ages, races and countries. The outbreak has taken millions of lives and has dramatically affected all aspects of our lives from health to economic. Although different platforms of vaccines have been developed and administered globally, not all residents have been fortunate to receive them. The more transmittable the virus becomes, the harder it is to achieve community-level protection. Two distinct kinds of advantages can be obtained from immunizing individuals. For any one person, the vaccine does protect against getting the illness, and it protects against severe illness, reduces hospitalization and decreases death rate. Additionally, community protection, sometimes referred to as herd immunity, is also achieved. More specifically, herd immunity refers to many people with an increased level of individual protection that the virus cannot find enough susceptible new individuals to infect and gradually diminishes, eventually disappearing in circulation in the community. To date, only about 51% of people worldwide have received their first dose, and nearly 40% have received the second dose, which indicates slow vaccination rates. It will take vaccination rates of about 70–85% to reach the herd immunity threshold and bring this pandemic to an end. If we do not reach the herd immunity threshold by next winter, COVID-19 may resurface as a seasonal virus. Due to the limited capacity of production of the vaccine in some countries and fast transmission rates, an important concern is the incidence of new viral mutant strains and their resistance to current vaccines. To meet global demands, vaccine production technology can be transported from developed countries to developing countries. Oxford AstraZeneca has worked with countries such as India and more recently with South Korea in this effort. Also, health education leaders are facing a serious challenge regarding vaccine hesitancy. It will be difficult for people who do not have sufficient knowledge of health systems to accept that an attenuated virus is to be injected into their body in the form of a vaccine; therefore, it presents resistance to vaccination. To overcome this challenge, educating the public about vaccines is essential. Another important issue to consider is that a percentage of breakthrough cases have been reported, highlighting the importance of having efficient therapeutic strategies for dealing with infected patients. It is likely that we will encounter a virus with greater lethality and wider spread in the future. It goes without saying that the capacity to produce modern vaccines, to help advance the production of nanoparticle-based vaccines and provide general knowledge to international communities must be prioritized by world governments. Hence, in the current scenario, we still need to rely on preventive measures as well as smart therapeutic plans. Along with the application of repurposed drugs used to manage COVID-19, the development of specific anti-COVID-19 drugs is necessary. In case the virus is not eradicated, therapeutic strategies will remain a pivotal tool for the future.

**Executive summary**

**Epidemiology**

- The coronavirus is a kind of virus that contains RNA genome and nucleocapsid phosphoprotein, which causes many respiratory diseases in humans and various ranges of diseases in animals.
- The epidemiological trends in the outbreak and fatality rate of COVID-19 vary from day to day, and new mutants of the virus were reported.

**Antiviral agents**

- Among the antiviral agents used in managing the COVID-19 crisis, favipiravir as a purine nucleic acid analog is used to shorten the clearance time of the virus from the body. Also, remdesivir was shown to be capable of inhibiting the virus with higher sensitivity and lower concentration.

**Antibiotics**

- Azithromycin is a macrolide that is effective in both Gram-negative and Gram-positive bacteria and is used to treat respiratory tract infections. However, to date, there is no reliable clinical proof for the success of azithromycin in COVID-19.

**Antiparasitic agents**

- Among the antiparasitic agents used in COVID-19, ivermectin can reduce the infectivity of coronavirus through binding to imp-α and imp-β1 proteins.

**Nonsteroidal anti-inflammatory drugs**

- Ibuprofen and naproxen as NSAIDs are used to suppress inflammation, fever and pain in the COVID-19 crisis. The virus must bind to angiotensin-converting enzyme 2 receptors to enter eukaryotic cells. These drugs inhibit the virus from attaching to the receptor. However, there is a great deal of disagreement about NSAIDs to improve patients' condition.

**Mucolytic agents**

- Among the mucolytic agents used in COVID-19, N-acetylcysteine and bromhexine help reduce mucus secretion. N-acetylcysteine leads to a reduction in disulfide bonds. These drugs also prevent the attachment of viruses to the angiotensin-converting enzyme 2 receptors.

**Immunomodulatory drugs**

- The administration of immune modulators could be a promising approach to circumvent harmful immunological responses. For instance, anakinra as an IL-1 antagonist is an effective immune modulator that can eliminate the requirement for mechanical ventilation in COVID-19 patients.

**Corticosteroids**

- It is supposed that keeping a balance between immune response and viral replication and infection should be considered. So, the administration of corticosteroids may be beneficial in avoiding cytokin storm. However, there are some controversy regarding the use of corticosteroids in COVID-19 patients.

**Immunosuppressant drugs**

- Among the immunosuppressant drugs, fingolimod can influence the aggregation and distribution of lymphocytes. Moreover, leflunomide can shorten the duration of virus shedding and hospitalization.

**Monoclonal antibodies**

- To suppress the release of cytokine storm and activation of immune responses, monoclonal antibodies are used to bind to the pivotal inflammatory cytokines and inhibit organ damage. For example, tocilizumab and sarilumab as inhibitors of IL-6 can be promising options in alleviating symptoms in COVID-19 patients.

**Anticoagulants**

- The activation of innate immunity during viral replication leads to the activation of immune-thrombosis. Therefore, it is thought that anticoagulants such as low molecular weight heparin and rivaroxaban can reduce the risk of thrombosis while enhancing microcirculation and preventing organ damage.

**Cardioprotective agents**

- The administration of some cardioprotective agents, such as aspirin and statins, before hospitalization can reduce mortality rates from acute lung injury and acute respiratory distress syndrome.

**Other treatments**

- It is suggested that SARS-CoV-2 can damage hemoglobin molecules. Hence, iron overload would be a common consequence of SARS-CoV-2 infection. Therefore, deferoxamine, which acts as a chelating agent may benefit these patients. Also, deferoxamine can reduce viral replication in the SARS-CoV-2 replication process. However, randomized, controlled trials are required in the management of COVID-19 patients.

**Mesenchymal stem cells**

- The possible mechanism of mesenchymal stem cell therapy in COVID-19 management would be through stimulation of the differentiation process and its immunomodulatory effects. Also, it could induce tissue repair through various mechanisms, including the secretion of anti-inflammatory cytokines, angiogenesis induction, immunomodulatory potential and extracellular vesicle release.
